# ESCO2’s oncogenic role in human tumors: a pan-cancer analysis and experimental validation

**DOI:** 10.1186/s12885-024-12213-w

**Published:** 2024-04-11

**Authors:** Yue Huang, Dapeng Chen, Yi Bai, Yamin Zhang, Zhiwen Zheng, Qingfeng Fu, Bocun Yi, Yuchen Jiang, Zhihong Zhang, Jianqiang Zhu

**Affiliations:** 1 Tianjin Institute of Urology, The Second Hospital of Tianjin Medical University, Tianjin, China; 2 Tianjin First Central Hospital Clinic Institute, Tianjin Medical University, Tianjin, 300192 China; 3 Department of Hepatobiliary Surgery, Tianjin First Central Hospital, School of Medicine, Nankai University, Tianjin, 300192 China

**Keywords:** Pan-cancer, ESCO2, Cell cycle, Single-cell

## Abstract

**Purpose:**

Establishment of sister chromatid cohesion N-acetyltransferase 2 (ESCO2) is involved in the mitotic S-phase adhesins acetylation and is responsible for bridging two sister chromatids. However, present ESCO2 cancer research is limited to a few cancers. No systematic pan-cancer analysis has been conducted to investigate its role in diagnosis, prognosis, and effector function.

**Methods:**

We thoroughly examined the ESCO2 carcinogenesis in pan-cancer by combining public databases such as The Cancer Genome Atlas (TCGA), Genotype-Tissue Expression Project (GTEx), UALCAN and Tumor Immune Single-cell Hub (TISCH). The analysis includes differential expression analysis, survival analysis, cellular effector function, gene mutation, single cell analysis, and tumor immune cell infiltration. Furthermore, we confirmed ESCO2’s impacts on clear cell renal cell carcinoma (ccRCC) cells’ proliferative and invasive capacities in vitro.

**Results:**

In our study, 30 of 33 cancer types exhibited considerably greater levels of ESCO2 expression in tumor tissue using TCGA and GTEx databases, whereas acute myeloid leukemia (LAML) exhibited significantly lower levels. Kaplan-Meier survival analyses in adrenocortical carcinoma (ACC), kidney chromophobe (KICH), kidney renal clear cell carcinoma (KIRC), kidney renal papillary cell carcinoma (KIRP), brain lower grade glioma (LGG), liver hepatocellular carcinoma (LIHC), lung adenocarcinoma (LUAD), mesothelioma (MESO), and pancreatic adenocarcinoma (PAAD) demonstrated that tumor patients with high ESCO2 expression have short survival periods. However, in thymoma (THYM), colon adenocarcinoma (COAD) and rectum adenocarcinoma (READ), ESCO2 was a favorable prognostic factor. Moreover, ESCO2 expression positively correlates with tumor stage and tumor size in several cancers, including LIHC, KIRC, KIRP and LUAD. Function analysis revealed that ESCO2 participates in mitosis, cell cycle, DNA damage repair, and other processes. CDK1 was identified as a downstream gene regulated by ESCO2. Furthermore, ESCO2 might also be implicated in immune cell infiltration. Finally, ESCO2’S knockdown significantly inhibited the A498 and T24 cells’ proliferation, invasion, and migration.

**Conclusions:**

In conclusion, ESCO2 is a possible pan-cancer biomarker and oncogene that can reliably predict the prognosis of cancer patients. ESCO2 was also implicated in the cell cycle and proliferation regulation. In a nutshell, ESCO2 is a therapeutically viable and dependable target.

**Supplementary Information:**

The online version contains supplementary material available at 10.1186/s12885-024-12213-w.

## Introduction

Cancer in most nations gradually displaces cardiovascular disease as the leading cause of premature mortality [1]. According to statistics, there will be 19.3 million new cancer cases and almost 10 million cancer-related deaths in 2020. The global cancer burden is expected to reach 28.4 million patients by 2040 [2]. Cancer treatment has steadily transitioned into the era of precision therapy because of the advanced high-throughput sequencing technology. Advanced cancer patients frequently receive targeted therapy and immunotherapy in the clinic. For instance, palbociclib (a CDK4/6 inhibitor) in conjunction with letrozole (24.8 months) resulted in considerably longer progression-free survival than letrozole alone (14.5 months) in patients with previously untreated ER-positive, HER2-negative advanced breast cancer [3]. Due to the identification of molecular targets and the growing understanding of their cellular effects, small molecule inhibitors have been created as a primary therapeutic approach for cancer treatment. The discovery of new molecular targets may contribute to advancing combined therapies, eradicating drug resistance, and increasing therapeutic efficacy. Small molecule inhibitors have a bright future; thus, scientists should investigate novel biomarkers to find potential therapeutic targets.

The cell cycle is a tightly controlled process that supports genetic material replication and cell proliferation. One of the distinguishing characteristics of malignancies is abnormal cell proliferation brought on by cell cycle dysregulation [4]. Dysregulation of the cancer cell cycle facilitates cell proliferation, which is driven by excessive mitotic signaling, inhibitory checkpoint failure, or both. Scientists have demonstrated that it is effective to target proteins associated with the cell cycle in order to limit tumor growth. The most notable successes in targeting cell cycle mechanisms have been inhibitors of CDK4 and CDK6 [5–7]. The introduction of these CDK4/6 inhibitors for clinical use constitutes a milestone in the treatment of breast cancer and may have broad ramifications for the management of other tumor types [8]. Despite the current success of CDK4/6 inhibitors, cancer therapy that targets cell cycle proteins is still in its infancy. An in-depth exploration of cell cycle regulatory mechanisms and their role in cancer will guide current cancer treatments and identify new therapeutic targets.

Establishment of sister chromatid cohesion N-acetyltransferase 2 (ESCO2) was identified as an effective target for cancer therapy, which is a pivotal protein in the cell division process [9]. ESCO2, the human homolog of yeast ECO1, works on proliferating cell nuclear antigen (PCNA) to stimulate sister chromatid cohesion [10, 11]. Acetylation of the SMC3 subunit of the adhesive protein via ESCO2 acetyltransferase facilitates sister chromatid cohesion, inhibiting cohesin release from chromatin [12]. The cohesin is a multiprotein complex whose typical role is to bind sister chromatids from S-phase to anaphase to prevent premature segregation of sister chromatids and to ensure equal segregation of chromosomes [13, 14]. Hence, the presence of ESCO2 ensures correct chromosomal segregation and makes recombinant DNA repair possible. Recent research has demonstrated that ESCO2 is strongly linked to the formation of several cancer types [15–19]. ESCO2 knockdown in human gastric cancer cell lines in vitro significantly inhibited cell proliferation and induced apoptosis by regulating P53 [17]. Meanwhile, ESCO2 can promote LUAD cell proliferation and metastasis by promoting aerobic glycolysis [16]. Similarly, Fu et al. identified that 53BP1-MDS ring-like structure disruption caused by ESCO2 depletion in colorectal cancer cells, which reduced the effectiveness of non-homologous end joining (NHEJ) repair and made cancer cells more susceptible to chemotherapy [20]. However, Guo et al. discovered that ESCO2 overexpression in colon cancer reduced MMP2’s transcriptional activity to limit tumor metastasis [19]. ESCO2’s involvement in cancer is therefore controversial. Investigating ESCO2’s biological function in other malignancies is necessary. A pan-cancer analysis can investigate the oncogene in several malignancies, allowing oncogenes’ effector function and differential expression analyses.

We identified ESCO2’s abnormal expression value in tumor and normal samples by pan-cancer analysis and then validated its protein value in clinical tissues. Subsequently, we confirmed the value of ESCO2 in assessing prognosis and performed gene enrichment analysis, single cell analysis and immuno-infiltration analysis for ESCO2.Moreover, we conducted in vitro experiments to corroborate our bioinformatics findings. ESCO2 is a unique and useful biomarker and a prospective molecular target.

## Materials and methods

### Data preparation and differential expression analysis

The Cancer Genome Atlas (TCGA) database (https://portal.gdc.cancer.gov/), which has 11,315 total samples, was employed to download the bulk mRNA sequencing profiles of 33 distinct cancer samples and their corresponding normal tissues. All gene expression data were standardized using the Transcripts Per Kilobase of Exon Model per Million Mapped Reads (TPM) for all samples. In the meanwhile, we collected the relevant clinical information on cancer patients. The Progression Free Interval (PFI), Disease Free Interval (DFI), and Disease-Specific Survival (DSS) timings were retrieved from UCSC Xena(https://xena.ucsc.edu/), and the Overall Survival (OS) time was acquired from TCGA.

Then, using the TIMER [21] and Xena Shiny databases (https://shiny.hiplot.cn/ucsc-xena-shiny/)-powerful online platforms that incorporated mRNA sequencing data from the TCGA and GTEx datasets [22]-we analyzed the ESCO2’s mRNA expression value between tumor and normal samples for 33 types of cancer. Furthermore, the ESCO2’s protein level was determined using the UALCAN online platform (https://ualcan.path.uab.edu/index.html). Finally, three pairs of paraffin-embedded kidney renal clear cell carcinoma (KIRC), bladder urothelial carcinoma (BLCA), and adjacent samples were collected from the Second Hospital of Tianjin Medical University. The pathologic type of these individuals was clear cell renal cell carcinoma (ccRCC, i.e., KIRC) or BLCA, and none of them received preoperative chemotherapy. More crucially, all patients have approved the use of the surgical material for academic research and publications. All methods were approved by The Institutional Review Committee and the Medical Ethics Committee of the Second Hospital of Tianjin Medical University. Written informed consents have been obtained from all subjects.

### Survival analysis and relationship with clinical stage

We filtered the cancer patients to assess the ESCO2’s impacts on the cancer cases’ prognosis. (a) The average value of several samples from the same patient was thought to represent the ESCO2 expression level. (b) cancer patients with a follow-up time of zero were excluded. After that, the survival R package was applied to run the survival analysis. The survfit function was utilized to model the Kaplan-Meier survival curve. The best-cutoff point was discovered using the “surv cutpoint” function in a survminer R package, which can repeatedly check all viable cutting points to get the highest rank statistic. We next divided each cancer type’s patients into two groups using the appropriate cutoff point for gene expression. The Kaplan-Meier survival curves were compared based on the two-sided long-rank test. Moreover, we performed Receiver operating characteristic curve (ROC) testing to assess the ESCO2’s ability to predict cancer across all types. Then, we investigated the relationship between ESCO2’s expression level and clinical parameters, including tumor size, stage, and grade.

### Functional enrichment analysis of ESCO2

We extracted the RNA sequencing profiles of cancer types where ESCO2 has prognostic value to investigate ESCO2’s carcinogenesis in malignancies. Hence, 12 cancer types, including thymoma (THYM), colon adenocarcinoma (COAD), rectum adenocarcinoma (READ), adrenocortical carcinoma (ACC), kidney chromophobe (KICH), KIRC, kidney renal papillary cell carcinoma (KIRP), brain lower grade glioma (LGG), liver hepatocellular carcinoma (LIHC), lung adenocarcinoma (LUAD), mesothelioma (MESO), and pancreatic adenocarcinoma (PAAD), were rolled into the next analysis. For each cancer type, individuals were grouped into ESCO2-high and ESCO2-low subgroups relied on the median value of ESCO2, respectively. After that, gene set enriched in two groups were detected by gene set enrichment analysis (GSEA). Gene sets, the 50 hall markers, were obtained from MSigDB [23] (https://www.gsea-msigdb.org/gsea/msigdb).

### The hunt for ESCO2-related regulatory genes

The above data shows that ESCO2 is a negative oncogene in most cancers. Therefore, we extracted the RNA-seq matrix from KICH, KIRC, LGG, LIHC, LUAD, and PAAD to study ESCO2-related regulatory genes. ESCO2 had a negative impact on these cancer types, which also accounted for more than 150 cases. Similarly, we split patients into ESCO2-high and ESCO2-low groups based on the median value of ESCO2 in each cancer type. Following that, differentially expressed genes (DEGs) between ESCO2-high and the ESCO2-low group were performed using limma R packge [24](*p* < 0.05, Log FC > 1). After identifying the intersecting genes of DEGs in each cancer, we calculated the association between ESCO2 and these DEGs by spearman’s correlation analysis for each cancer type, respectively (*p* < 0.05, Cor > 0.4). Intersecting genes for these highly correlated genes were considered ESCO2-related regulatory genes. Then, using the clusterProfiler R [25] package, the Kyoto Encyclopedia of Genes and Genomes (KEGG) [26–28] and Gene Ontology (GO) pathway enrichment analyses were performed on ESCO2-related regulatory genes. Incorporating ESCO2-related regulatory genes into the String database (https://cn.string-db.org/) to form a PPI network enables the identification of core genes.

### Single-cell analysis of ESCO2 in cancers

Tumor Immune Single-cell Hub (TISCH, http://tisch.comp-genomics.org/home/) is a robust database created for multiple single-cell analyses containing nearly 190 single-cell databases. We estimated the ESCO2 expression level across multiple cancer types in each cell type. Our study focused on the distribution of ESCO2 in KIRC cell subpopulations. The expression levels of ESCO2 in each cell type were quantified and visualized by a heatmap, scatter diagrams, and violin plots. In addition to the single-cell dataset of KIRC contained in the TISCH database, we also downloaded additional single-cell sequencing data from GSE156632 [29] in the Gene Expression Omnibus (GEO) database to validate ESCO2 expression level in each cell types.

### Cell culture and siRNA transfection

The Cell Resource Center Affiliated with the Chinese Academy of Medical Sciences provided the human ccRCC cell lines A498 and human BLCA cell lines T24. In a 5% CO_2_ humidified cell incubator at 37 °C, A498 and T24 cells were grown in Dulbecco’s modified Eagle medium (DMEM) (Gibco BRL Life Technologies Inc., USA) with 10% FBS (Gibco BRL Life Technologies Inc., USA) and 1% penicillin-streptomycin (Hy-clone, CA, USA).

Small interfering RNAs (siRNAs) were ordered from GenePharma. The si-NC and si-ESCO2 siRNA were transfected into A498 and T24 cells with a Transfection reagent. When cells occupied 70–80% of a 50 mm Petri dish, transfection experiments were conducted; the whole medium was aspirated, and 2 mL of opti-MEM was applied to each well. The siRNA (GenePharma, Shanghai, China) and Transfection reagent (Cat#:L3000075, Thermo Fisher Scientific, United States) were added to 500 µL opti-MEM, mixed, and incubated for 20 min at room temperature. After that, the siRNA-Transfection reagent mixture was added to each well. Each well was switched to a complete medium 6 h after the transfection assay. The sequences for ESCO2 siRNA are as follows: si-ESCO2-1 (forward: GCAAAUCAAGGCUCACCAUTT; reverse: AUGGUGAGCCUUGAUUUGCTT) and si-ESCO2-2 (forward: CUCUUAGACCAGGAUUAUCTT; reverse: GAUAAUCCUGGUCUAAGAGUG).

### qPCR and western blotting analysis

After the cells were transfected for 48 and 72 h, we harvested cells for RNA and protein extraction, respectively. Following the protocol, the total RNAs of cells were extracted using TRIzol reagents (Invitrogen). We then used cDNA reverse transcription kits to synthesize cDNA, and the mRNA expression level was calculated through qPCR assay in q225. We used the 2^−*ΔΔ*Ct^ method to calculate GAPDH as an internal reference for mRNA. The ESCO2’s sequences of primers are as follows: F, CACTGGGACGCACCCAAAA, R, CACTTGCCTTGTCGCAAAAG (Sangon biotech). The following are the primer sequences for the GAPDH gene.: F, CAGGAGGCATTGCTGATGAT, R, GAAGGCTGGGGCTCATTT (Sangon biotech).

The tissues or cells were collected and lysed in RIPA lysis buffer (Cat#:89,901, Thermo Fisher Scientific, USA) with supplementation of a protease inhibitor cocktail (Cat#:539,131, Roche, Switzerland) in the ice for 30 min. The total protein extracts were measured by the ABC method (Cat#:23,227, Thermo Fisher Scientific, USA), separated by 10% sodium dodecyl sulfate-polyacrylamide gel electrophoresis (SDS-PAGE), and transferred to the nitrocellulose membrane. The membrane was blocked using 5% non-fat milk or 5% BSA (Solarbio, Beijing, China), then incubated with primary antibodies anti- (ESCO2,Cat#:23525-1-AP, diluted 1:2000, Proteintech, USA; CDK1, Cat#:23525-1-AP, diluted 1:2000, Proteintech, USA). The proper secondary antibody is chosen based on the primary antibody’s type, including goat anti-mouse HRP-conjugated IgG (Cat#:SA00001-1, 1:2000 dilutions, Proteintech, USA) and goat anti-rabbit HRP-conjugated IgG (Cat#:SA00001-2, 1:2000 dilutions, Proteintech, USA). The target band signals were captured with the help of a BIO-RAD ChemiDoc XRS chemiluminescence system (Bio-Rad Inc., CA, USA). The densitometry of all Western blots was analysed using a Gel-Pro analyzer (Media Cybernetics, USA). Imagej software was used to perform quantitative analysis with β-actin as a control (1.52a, National Institutes of Health, Bethesda, MD, USA).

### Cell proliferation and colony formation assay

Briefly, 2000 cells per well were planted onto 96-well plates for the cell proliferation assay, and transfection assay was conducted after cell fixation. Cells were cultured in the incubator for 12, 24, 48, and 72 h. After that, each well received 10 µL of Cell Counting Kit-8 reaction solution (CCK8, Cat#:CA1210, Solarbio Science & Technology Co., Ltd., Beijing, China) and was incubated for 1 h. The light absorption values (OD) for si-NC and si-ESCO2 groups were captured at 450 nm on a Varioskan Flash Multimode plate reader (Thermo Fisher Scientific Inc., USA).

Cancer cells per well were seeded onto a 12-well plate, and the medium was changed every two days. After being stained with 0.5% crystal violet (Cat#: C0121, Beyotime) for 20 min and fixed with 4% paraformaldehyde at room temperature for 10 min, colonies were counted 14 days later.

### Ethynyl-2-Deoxyuridine (EdU) incorporation assay

Cancer cells were seeded in 3.5 cm confocal dishes (Thermo Fisher Scientific Inc., USA) with 50,000 cells per dish. An appropriate amount of 50 µM EdU (Cat#:K1077, ApexBio Technology LLC) medium was prepared by diluting the EdU solution with cell culture medium at a ratio of 1000:1.1 ml of 50 µM EdU medium was added after the entire medium had been aspirated, and it was then incubated for 2 h. After discarding the medium, it was washed with PBS, fixed with 4% paraformaldehyde, and then permeabilized with 0.5% TritonX-100 (Cat#:X100, Sigma-Aldrich, USA). 200 μm of 1x Apollo (Cat#:K1077, ApexBio Technology LLC) staining reaction solution was added to each dish, incubated in the dark for 30 min. Nuclei were stained with DAPI (Cat#:MBD0015, Sigma-Aldrich, USA) for 10 min. Using a Nikon ECLIPSE 80i fluorescence microscope, images were captured, and ImageJ was used to count the cell number (1.52a, National Institutes of Health, Bethesda, MD, USA).

### Transwell migration and wound healing assay

Matrigel (BD Biosciences, Franklin Lakes, NJ, USA) was evenly spread in transwell chambers (Cat#: 3422, Corning Inc., Corning, NY, USA), and cells starved for 24 h were seeded in a serum-free medium in the upper chamber. Medium containing 10% FBS was added to the lower chamber as a catalyst. The upper chamber was removed and separated after roughly 24 h, then fixed with methanol for 10 min, rinsed with PBS, and stained with 0.1% crystal violet (Cat#:G1064, Solarbio Science & Technology Co., Ltd., Beijing, China). Under a microscope, cells that had been pierced were viewed and counted (CKX41, Olympus, Tokyo, Japan).

Cells were seeded uniformly in micro-insert 4-wells in a µ-dish^35 mm, high^ ibiTreat (ibidi GmbH, Germany). The culture inserts were removed when the cell density reached 90%. The 500 mm wide scratching gaps were washed with phosphate-buffered solution (PBS, Cat#:D1020, Solarbio Science & Technology Co., Ltd., Beijing, China) and were replaced with fresh medium. The dynamic changes of wells were recorded at 0, 12, 24 and 48 h on a microscope (Olympus, Japan).

### Statistical analysis

Using the Wilcox test, comparisons between the two groups were made. For analyzing three or more groups, the one-way ANOVA test was performed. For all statistical calculations, R studio and GraphPad software were used. The threshold for significance was defined at *P* < 0.05.

## Results

### ESCO2 expression level in various cancer

**Fig. 1:**
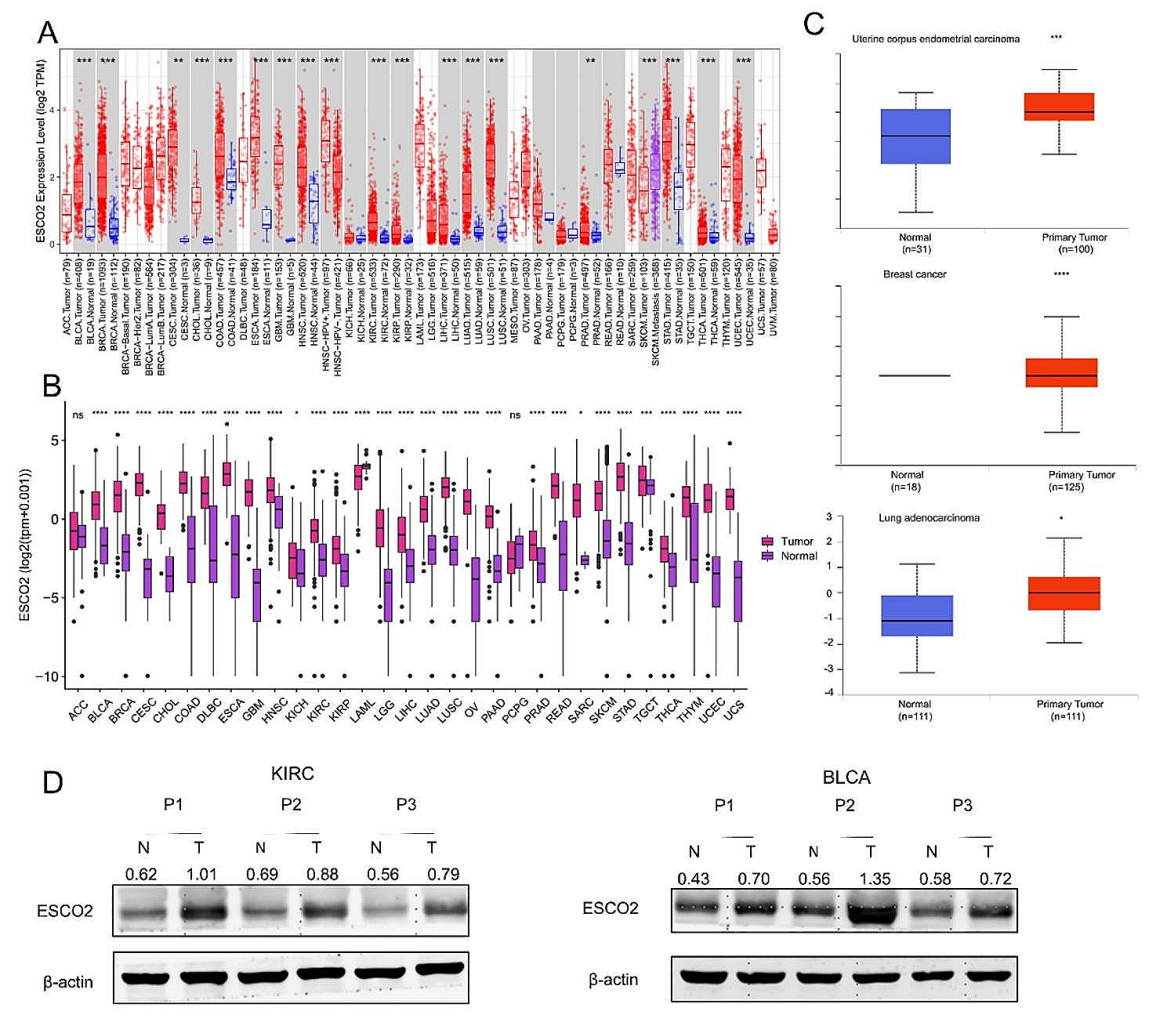
ESCO2 differential expression in pan-cancer. Comparison of ESCO2 expression between tumor and normal samples in TCGA database. Comparison of ESCO2 expression between tumor and normal samples in TCGA and GTEx database. (C) Comparison of ESCO2’s protein level between tumor and normal samples. (D) Western blot protein detection of the ESCO2 expression levels in adjacent normal tissues and paired cancer tissues, including KIRC and BLCA. The optical density ratio of bands represent objective proteins to band of β-actin was calculated

Typically, tumor tissue exhibits higher or lower oncogene expression levels than normal tissue. Using the TCGA database, our work found that ESCO2 is substantially expressed in the tumor tissue of most cancer types. However, the ESCO2 expression value did not significantly change in KICH, PAAD, pheochromocytoma and paraganglioma (PCPG), or READ (Fig. 1A). Considering only a few normal samples available in the TCGA database, we combined the TCGA and GTEx databases. 30 of 33 cancer types exhibited considerably greater levels of ESCO2 expression in tumor tissue, whereas acute myeloid leukemia (LAML) exhibited significantly lower levels. In the ACC and PCPG, the ESCO2 expression levels of the tumor and normal tissues were similar (Fig. 1B). ESCO2 expression was raised in most cancers, supporting its oncogene function.

Furthermore, we evaluated the ESCO2 protein levels. We only found significantly higher protein levels of ESCO2 in tumor tissues of uterine corpus endometrial carcinoma (UCEC), breast invasive carcinoma (BRCA), and LUAD due to the UALCAN database’s limited inclusion of cancer types (Fig. 1C). To further explore the alterations in ESCO2’s protein levels in carcinogenesis, we collected samples of KIRC and BLCA. In KIRC and BLCA, the protein level of tumor samples was noticeably greater than para-cancerous ones (Fig. 1D).

### ESCO2’s prognostic value across cancers

**Fig. 2:**
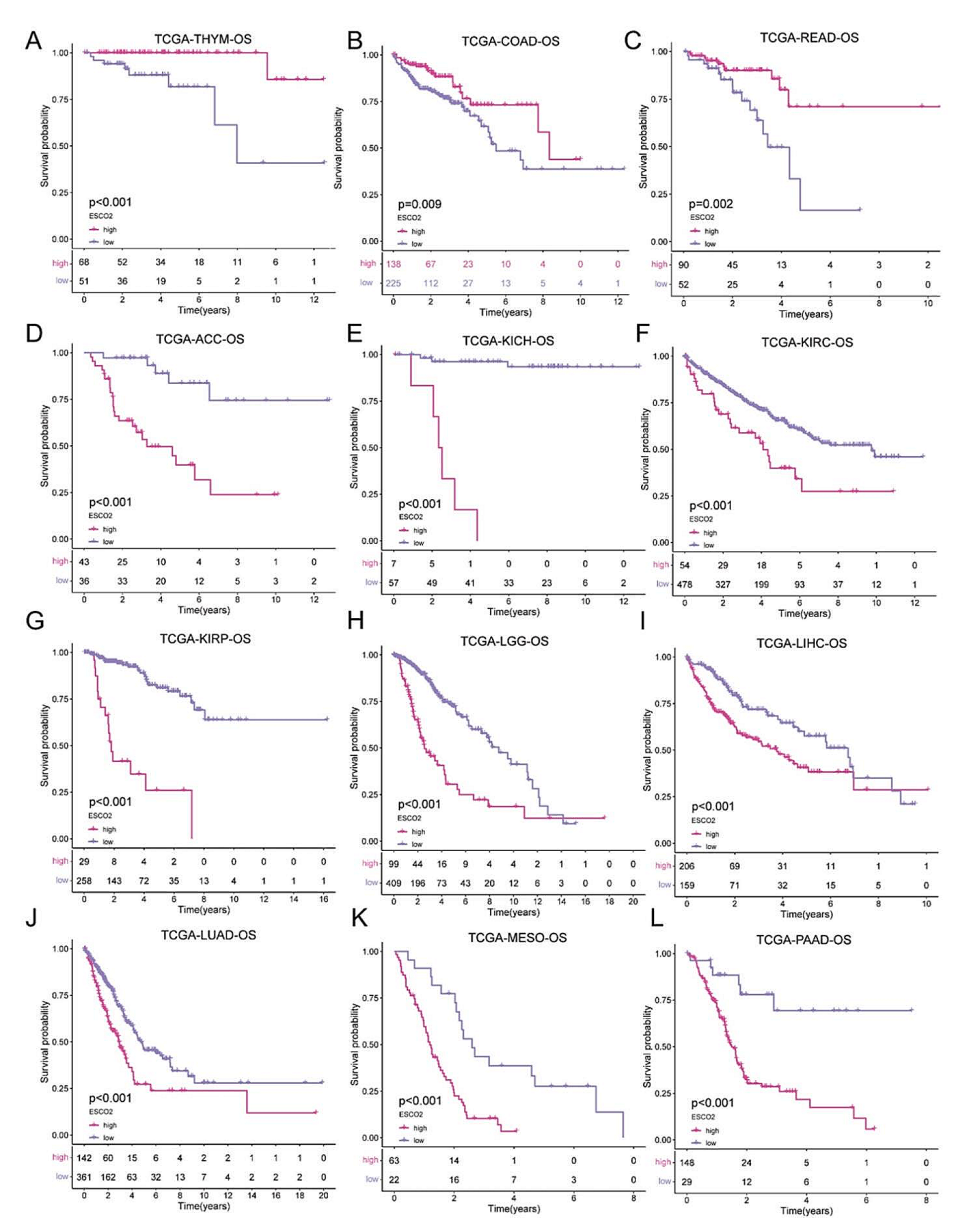
ESCO2’s Prognostic Value Across Cancers. (A–L) Kaplan–Meier analysis of the association between ESCO2 expression and OS

Using the TCGA database and focusing on OS, DFI, DSS, and PFI, we conducted a Kaplan-Meier survival analysis in each cancer to examine the association between ESCO2 and the cancer patient’s prognosis. Kaplan-Meier survival analyses in ACC, KICH, KIRC, KIRP, LGG, LIHC, LUAD, MESO, and PAAD demonstrated that tumor patients with high ESCO2 expression have short survival periods (Fig. 2D, E, F, G, H, I, J, K and L). While ESCO2’s expression was linked to better outcomes in patients with THYM, COAD, and READ (Fig. 2A, B and C). ROC analysis revealed that the ESCO2 performed well in predictive accuracy, with AUC values in ACC, KICH, KIRP, LGG, MESO, LIHC, and LUAD performing well (Figure S1A). High ESCO2 expression was linked to a worse prognosis in cancer patients with KIRP, LIHC, and PAAD, according to Kaplan-Meier survival analysis for DFI (Figure S1B). Similarly, as shown in Figures S2 and S3, cancer patients with increased ESCO2 expression had worse DSS and PFI across various malignancies. Our investigation revealed that ESCO2 might enhance tumor initiation and progression.

### ESCO2 was positively related to tumor progression

**Fig. 3:**
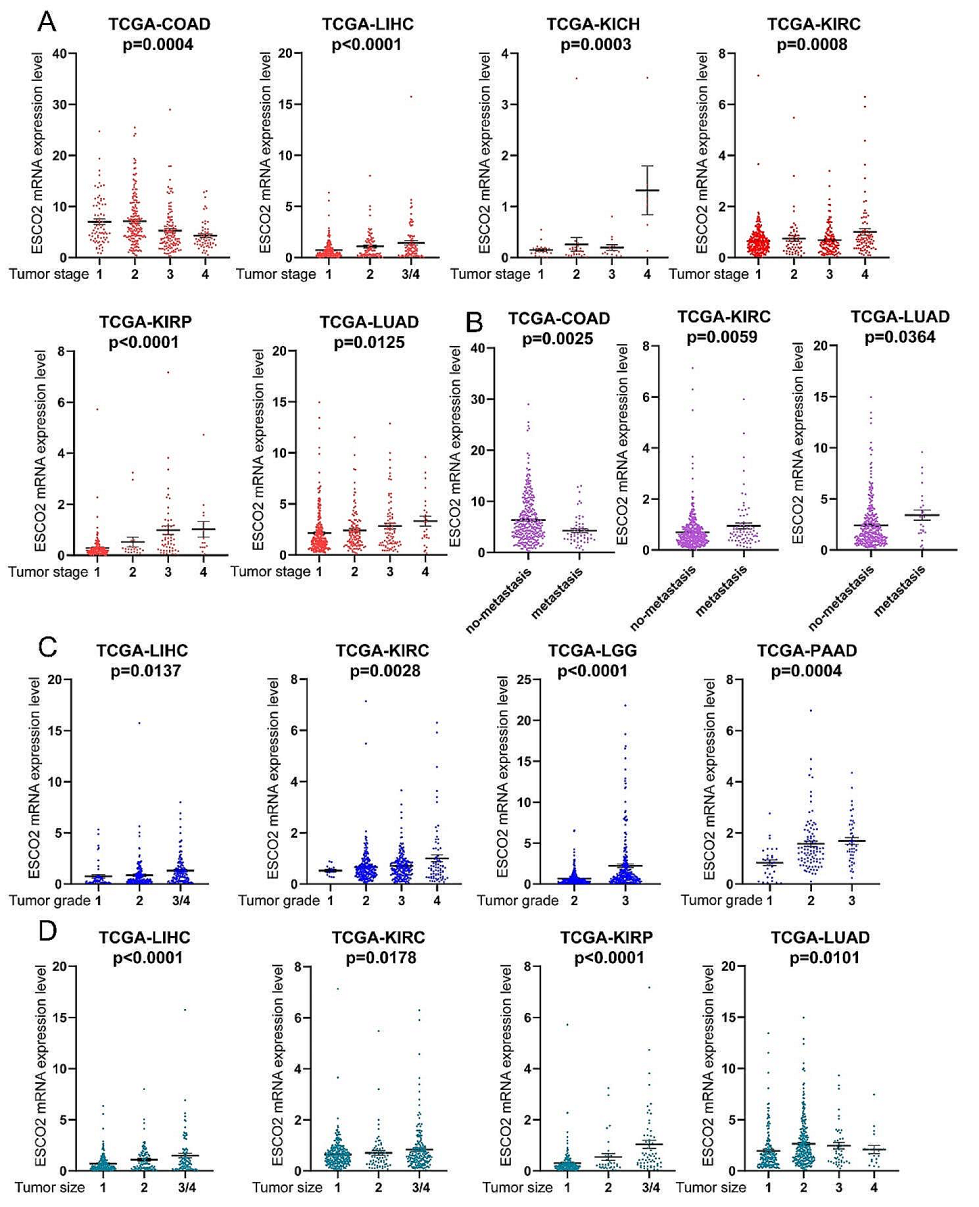
ESCO2 expression was positively correlated with tumor progression. Association between ESCO2 expression and tumor stage (A), tumor metastasis (B), tumor grade (C) and tumor size (D)

To confirm the ESCO2 role in cancer progression, a correlation analysis was performed between the ESCO2’s mRNA value and clinical features. We discovered that ESCO2 expression was significantly increased in advanced tumor stage in several malignancies, including KICH, KIRC, KIRP, LUAD, and LIHC. On the contrary, ESCO2 expression was higher in lower tumor stages than in higher tumor stages in COAD (Fig. 3A).

In LIHC, KIRC, LGG, and PAAD, ESCO2 expression was positively correlated with tumor grade (Fig. 3C). Similarly, ESCO2 may also affect the tumor size: Tumor size and ESCO2 expression were positively linked in LIHC, KIRC, KIRP, and LUAD. (Figs. 3D). Furthermore, ESCO2 was crucial for the metastasis of COAD, KIRC, and LUAD (Fig. 3B). Tumor stage, size, and metastasis are the most widely used clinical indicators for evaluating the progression of cancer. Our investigation verified the connection between ESCO2 and the aforementioned indicators, indicating that ESCO2 is involved in tumor progression.

### Analysis of ESCO2-related regulatory pathways

**Fig. 4:**
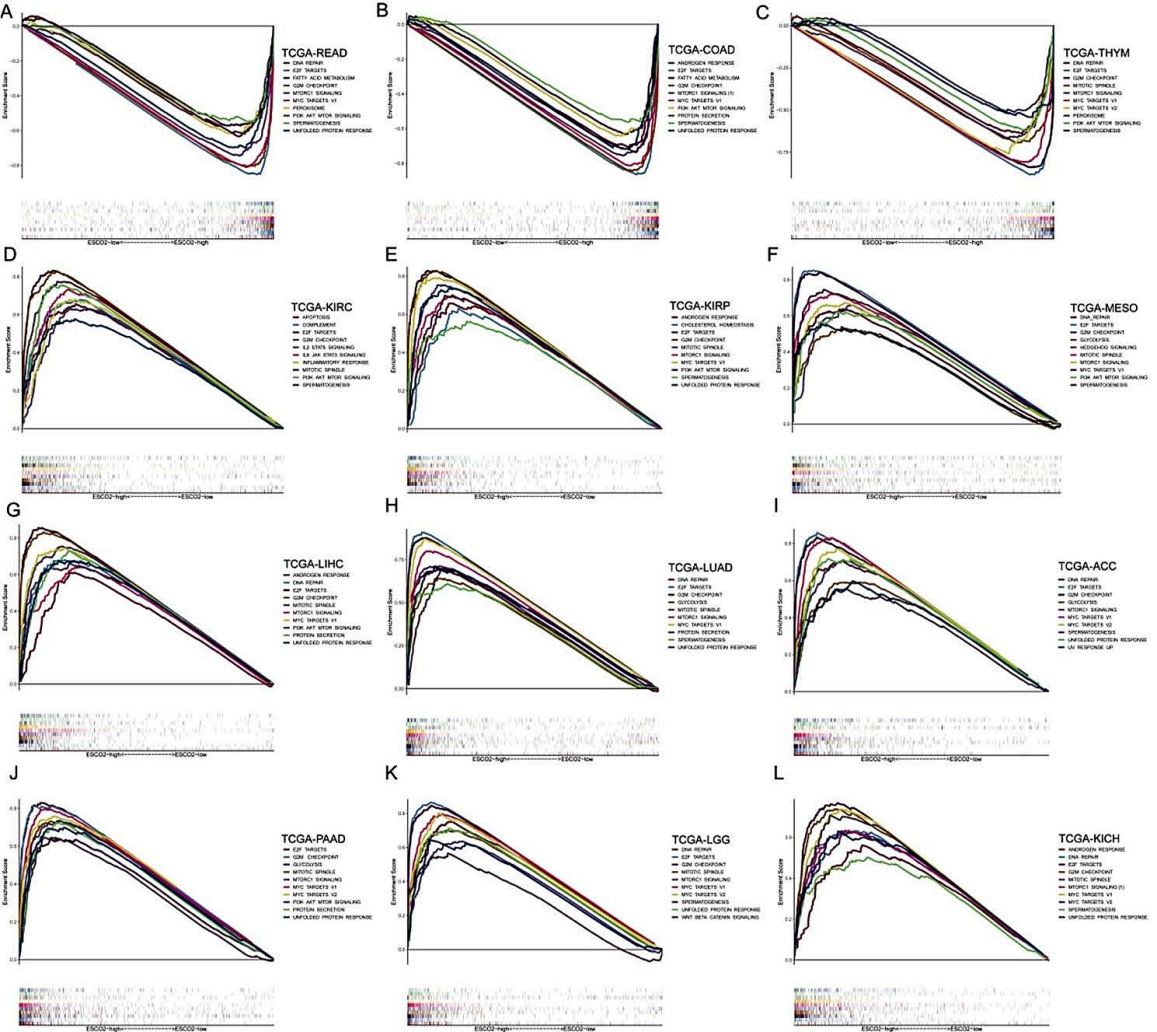
Results of gene set enrichment analysis (GSEA) based on the 50 hallmarks. (A–L) The GSEA analysis between ESCO2-high and ESCO2-low groups in READ, COAD, THYM, KIRC, KIRP, MESO, LIHC, LUAD, ACC, PAAD, LGG and KICH. Each panel’s left and right sides represent the enriched pathways of the ESCO2 high and low expression group, respectively

We gathered RNA sequencing profiles of cancer types where ESCO2 could significantly affect patients’ overall survival times to examine the ESCO2 oncogenic role in malignancies. For each cancer type described in the methods, cancer cases were grouped into ESCO2-high and -low subgroups according to the median value of ESCO2, respectively. Gene set enriched in two groups were then detected by gene set enrichment analysis. For visualization, we chose the top-10 NSE-ranked enriched pathways. Intriguingly, GSEA results were remarkably consistent across the 12 cancer types, indicating the validity of our findings (Fig. 4A, B, C, D, E, F, G, H, I, J, K and L). Genes upregulated in the ESCO2-high group showed the E2F targets enrichment, G2M checkpoint, mitotic spindle and Mtorc1 signaling, which were associated with cell cycle and proliferation. Meanwhile, these ESCO2-high patients exhibited highly enriched DNA repair. In addition to cell proliferation-related pathways, glycolysis and fatty acid metabolism pathways were enriched in the ESCO2-high group in several cancer types. Furthermore, ESCO2 seemed to participate in the modulation of cancer inflammation, such as IL2 STAT5 signaling.

**Fig. 5:**
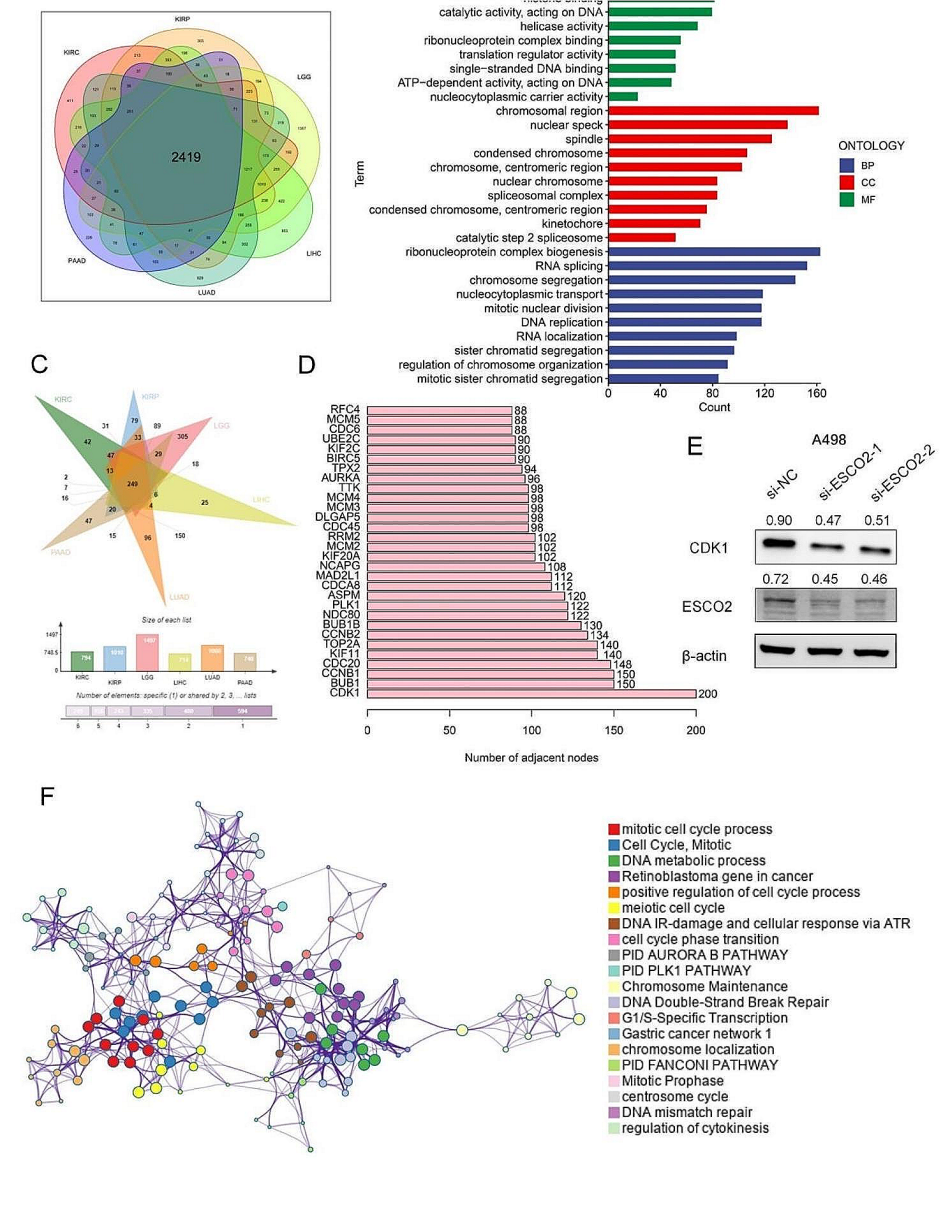
Analysis of ESCO2-related regulatory pathways. (A) The intersecting genes of DEGs between the ESCO2-high and ESCO2-low groups. (B) Gene Ontology analysis of the 2419 DEGs. FDR < 0.05 was considered significantly enriched. (C) The intersecting genes of DEGs with a high correlation to ESCO2 using spearman’s correlation analysis (*p* < 0.05, Cor > 0.4). (D) Bar plot of the top 30 genes with the largest number of adjacent nodes in the PPI network of ESCO2-related regulatory genes. (E) Western blot results manifested the down-regulated expression of CDK1 compared with the si‐con group in A498 cells.The optical density ratio of bands represent objective proteins to band of β-actin was calculated. (F) The ESCO2-related regulatory gene functions were mainly enriched in mitotic cell cycle and DNA metabolic processes. The interactive network was constructed using the meta scape online platform

To investigate ESCO2-related regulatory genes, we divided cancer patients into ESCO2-high and ESCO2-low groups based on the median ESCO2 value in six cancer types specified in the materials and methods. We then obtained 2419 intersecting genes, which mainly enriched on histone binding, ATP hydrolysis activity and helicase activity (Fig. 5A and B). The results of the GO analysis also confirmed that ESCO2 was primarily responsible for controlling cell growth. After spearman’s correlation analysis, we further identified the ESCO2-related regulatory genes. 249 ESCO2-related regulatory genes were collected in our study, which was highly correlated with ESCO2 across cancer types (Fig. 5C). These ESCO2-related regulatory genes were primarily involved in cell-division-related processes, like the mitotic cell cycle and DNA metabolic processes (Fig. 5F). We successfully identified the core genes by building the PPI network of ESCO2-related regulatory genes. The regulatory genes network’s nucleus is CDK1 (Fig. 5D). Cell cycle kinase family member Cyclin-dependent kinase 1 (CDK1) significantly impacts cell cycle progression [30, 31]. After the ESCO2 knockdown, we discovered that the CDK1 expression was significantly decreased (Fig. 5E). Therefore, CDK1 may be an important signaling pathway regulated by ESCO2 in KIRC.

### ESCO2 analysis at the single-cell level

**Fig. 6:**
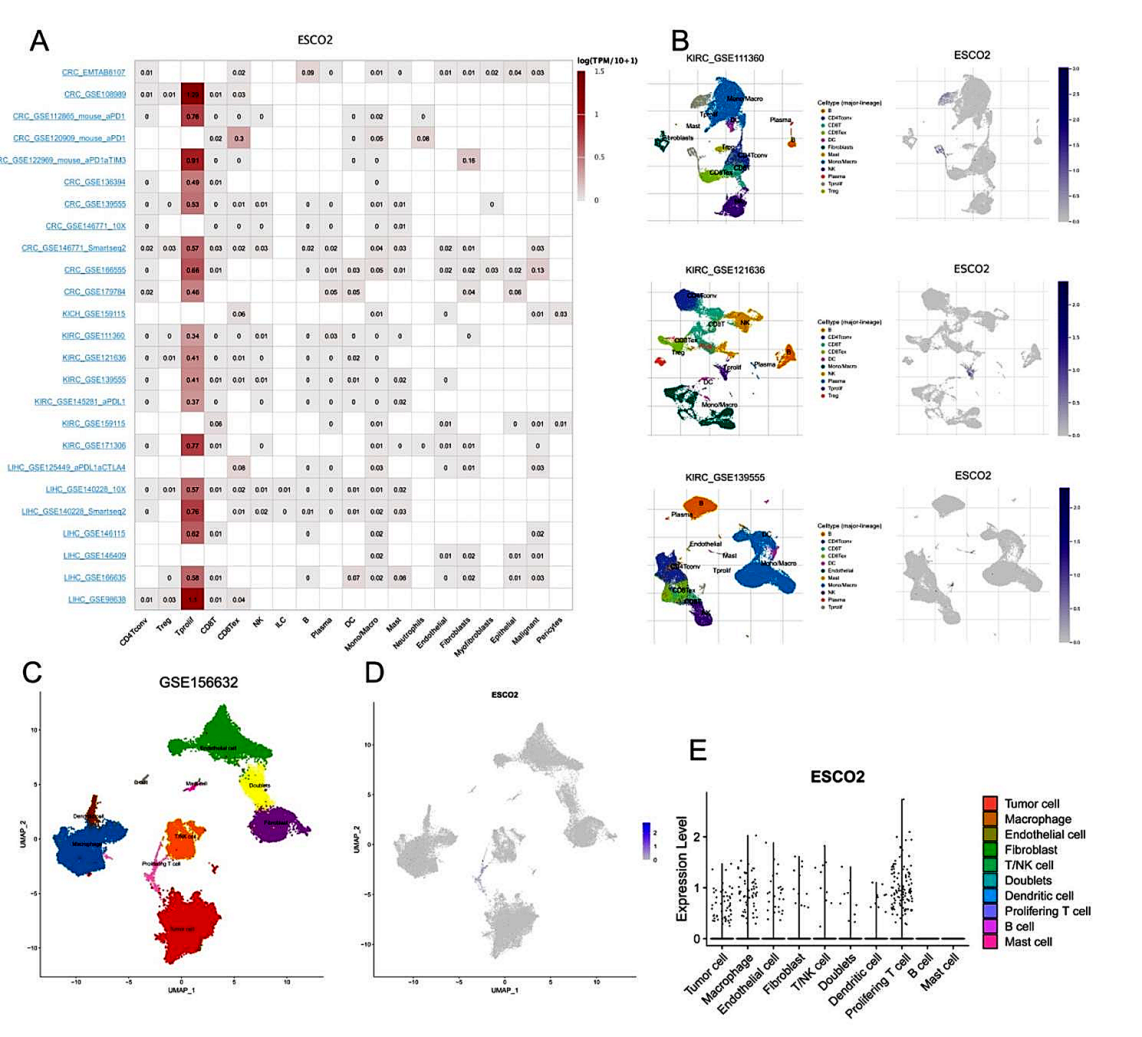
Single-Cell Analysis of ESCO2 in Cancers. (A) Summary of ESCO2 expression of 23 cell types in 31 single-cell datasets. (B) UMAP plots of all single cells of ccRCC patients, showing all cell types in the plot. (C) UMAP plot of all cell clusters in GSE156623 dataset. (D) UMAP plot and Violin plots(E) showing the expression of ESCO2 in each cell types

Recent studies have shown that cell cycle proteins play an additional role in tumor development, affecting not only tumor cells but also their microenvironment and modulating anti-tumor immune responses [32]. For instance, inhibition of CDK4/6 enhances tumor cell immunogenicity through multiple mechanisms [7]. Meanwhile, CDK4/6 inhibitors substantially reduce the proliferation of regulatory T cells and encourage cytotoxic T cells to eliminate tumor cells [33]. Therefore, we next explored the ESCO2’s role in the anti-tumor immune response. By scRNA-seq, we can investigate the ESCO2 at the single-cell level. Using single-cell sequencing of COAD, KIRC, KICH, LIHC and PAAD, we observed that ESCO2 was expressed not only in malignant cells but also in endothelial and immune cells, including T cells, macrophages, NK cells and B cells (Fig. 6A). Significantly, ESCO2 was strongly expressed in proliferating T cells. We found that ESCO2 was almost exclusively expressed in proliferating T cells in KIRC (Fig. 6B). To further validate the results observed in the TICSH database, we subjected all quality single cells of KIRC(GSE156632) to single-cell processing procedures. Similarly, ESCO2 was significantly expressed in proliferating T cells, but we observed a higher expression of ESCO2 in tumor cells and macrophages as well (Fig. 6C, D and E).

### ESCO2 Promotes Human ccRCC and BLCA cell proliferation and invasion in vitro

**Fig. 7:**
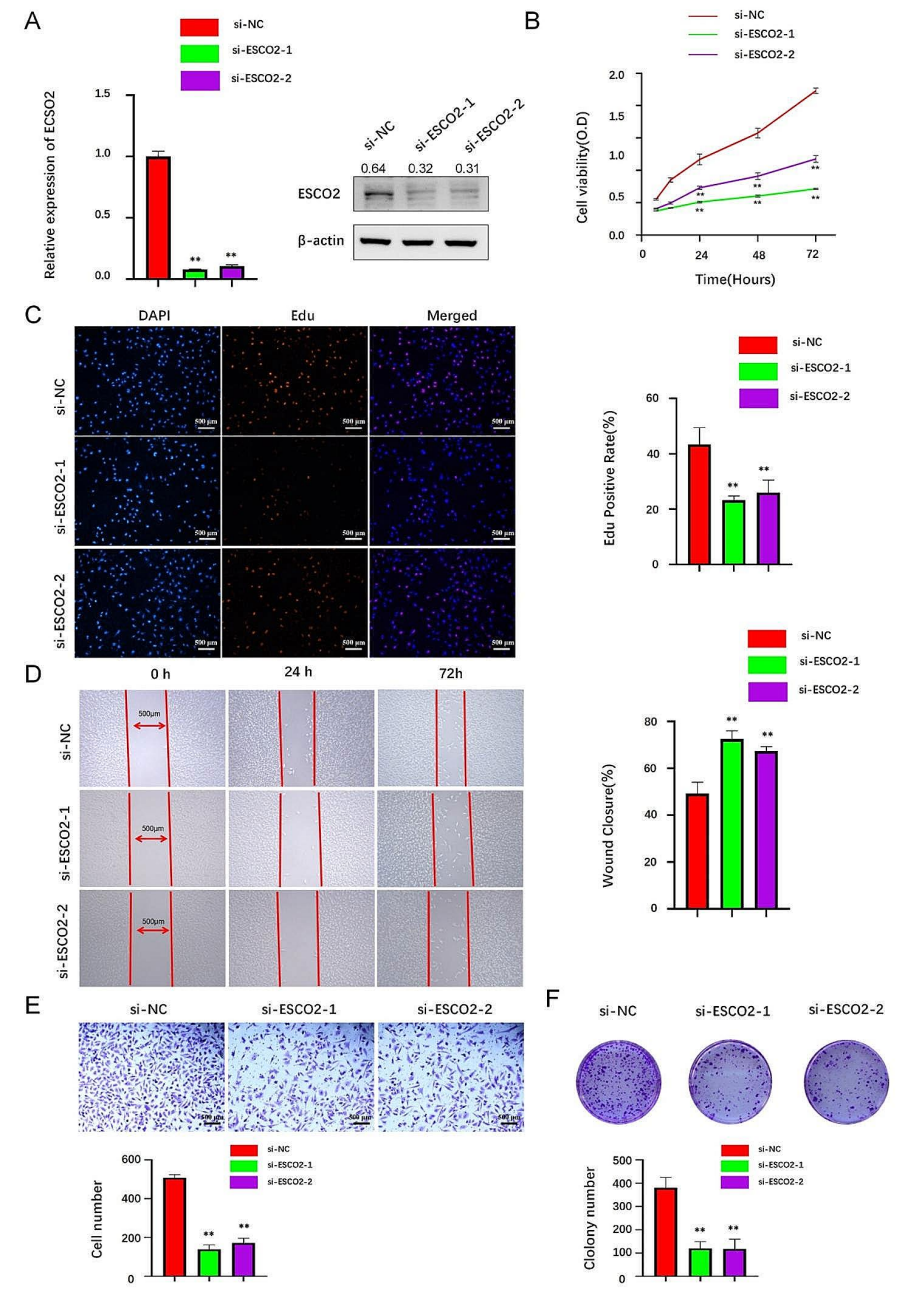
ESCO2 knockdown reduced cell proliferation, invasion, and migration in ccRCC. (A)A498 cells were transfected with si-ESCO2#1 and si-ESCO2#2, the level of ESCO2 was evaluated by qRT-PCR and Western blot. The optical density ratio of bands represent objective proteins to band of β-actin was calculated. (B) CCK8 analysis of cell viability in ESCO2-knockdown A498 cells at 0, 24, and 72 h, respectively, compared with the si‐con group. (C) EdU proliferation assay in A498 cells transduced with si-NC, si-ESCO2#1 or si-ESCO2#2. Scale bar = 500 μm. (D) Transwell and Wound Healing assay (E) revealed that ESCO2 silencing inhibited the invasion and migration ability. Scale bar = 500 μm. (F) ESCO2 silencing repressed colony formation in A498 cells. *: *P* < 0.05, **: *P* < 0.01, relative to untreated control

**Fig. 8:**
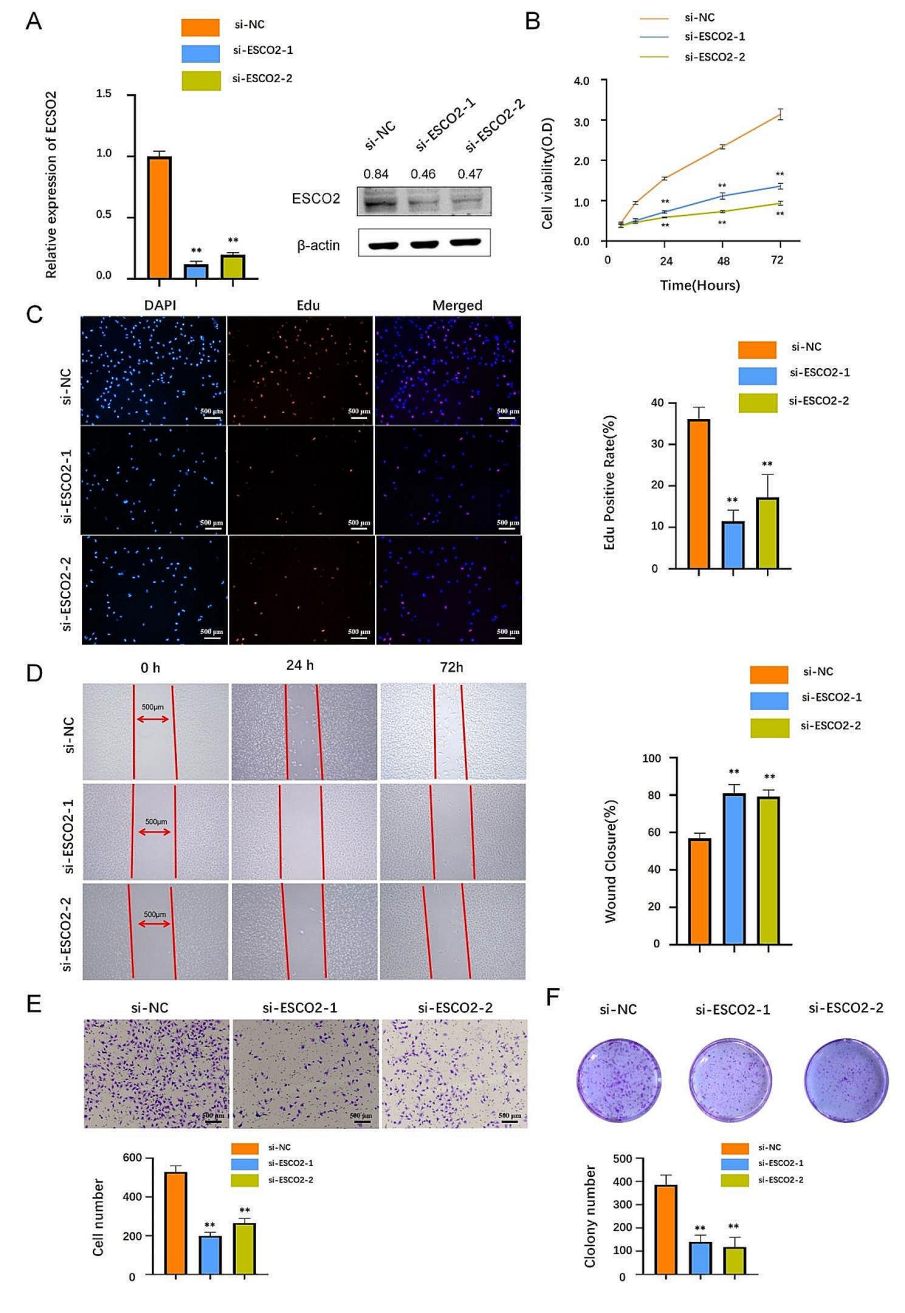
ESCO2 knockdown inhibited cell proliferation and invasion in BLCA cell lines. (A)T24 cells were transfected with si-ESCO2#1 and si-ESCO2#2, the level of ESCO2 was evaluated by qRT-PCR and Western blot. The optical density ratio of bands represent objective proteins to band of β-actin was calculated. (B) CCK8 analysis of cell viability in ESCO2-knockdown T24 cells at 0, 24, and 72 h, respectively, compared with the si‐con group. (C) EdU proliferation assay in T24 cells transduced with si-NC, si-ESCO2#1 or si-ESCO2#2. Scale bar = 500 μm. (D) Transwell and Wound Healing assay (E) revealed that ESCO2 silencing inhibited the invasion and migration ability. Scale bar = 500 μm. (F) ESCO2 silencing repressed colony formation in T24 cells

To confirm ESCO2’s biological effector role in carcinogenesis, we first knocked it down in human ccRCC cells. qt-PCR and Western blotting results showed that ESCO2 was successfully knocked down (Fig. 7A). Results from the CCK8 assay, colony formation assay, and EdU staining showed that the A498 cells’ ability to proliferate was significantly decreased by ESCO2 suppression than the control group (Fig. 7B, C and F). This is consistent with our previous bioinformatic analysis that ESCO2 significantly regulates cell cycle progression. To further identify the potential role of ESCO2 in the invasive and migration of A498 cells. According to the findings of Transwell and Wound Healing, silencing ESCO2 inhibits invasion and migration (Fig. 7D and E). To confirm ESCO2 as a potential pan-cancer biomarker, we further validated the carcinogenesis of ESCO2 in bladder cancer cell lines. According to the results of the experiments in vitro, the proliferative capacity of T24 cells was significantly inhibited after ESCO2’s knockdown (Fig. 8A, B, C and F). Furthermore, the invasive ability of the T24 cells after knockdown of ESCO2 significantly decreased compared with the control group (Fig. 8D and E). We confirmed, therefore, that ESCO2 is required for the proliferation and invasion of human ccRCC and BLCA cells.

## Discussion

Cancer treatment has evolved significantly, with the introduction of immunotherapy and targeted medicines increasing patient survival rates for those with advanced or metastatic cancer, but overall clinical outcomes remain disappointing. The challenge of treating advanced cancers has inspired researchers to explore the underlying mechanisms that contribute to cancer growth, which will help identify potentially effective therapeutic targets. Unquestionably, one of the most promising therapeutic targets is cell cycle related protein. Palbociclib, the first CDK4/6-specific inhibitor, was created in 2004 and has proven effective against a variety of human cancer cell lines [34]. All three CDK4/6 inhibitors (palbociclib, ribociclib, and abemaciclib) are currently approved by the United States Food and Drug Administration (FDA) for the treatment of breast cancer. In addition, numerous inhibitors of cell-cycle proteins, including CDK9, CDK2, and CDK5, are in clinical trials [35, 36]. Cell cycle regulating proteins such as CDCA4, CDCA8, and KIF2C have been linked to tumor growth and progression, affecting the proliferation, migration, invasion and metastasis of cancer cell lines [37–39]. ESCO2 was first reported in Roberts syndrome, whose inactivation mutation causes Roberts Syndrome [11]. Subsequent researchers have identified that ESCO2 is crucial in controlling cell mitosis and preserving genomic stability because it bridges two sister chromatids and is involved in the mitotic S-phase adhesins acetylation [40, 41]. As described in the context, current researchers consider ESCO2 an excellent therapeutic target as an oncogene that promotes cancer development. However, current cancer research on ESCO2 is limited to a few cancers, such as lung, stomach, and colon cancers. No studies are focusing on ESCO2 in multiple cancers that can shed light on the similarities of ESCO2 in cancer. In this study, we identified that ESCO2 was a reliable biomarker for cancer patients and could accurately predict the cancer patient’s prognosis. Furthermore, ESCO2 participated in mitosis, the cell cycle, DNA damage repair, other processes, and tumor immune infiltration. Finally, we confirmed that ESCO2 is essential for the proliferation and invasion of human ccRCC and BLCA cells in vitro.

First, we evaluated the ESCO2’s mRNA expression value in 33 cancers by TCGA and GTEx databases. The analysis showed that ESCO2 expression was upregulated in most cancer tissues except LAML, ACC and PCPG. Due to post-transcriptional processing, protein and mRNA gene expression levels may differ. Thus, we validated ESCO2 protein levels in KIRC and BLCA clinical samples. ESCO2 protein values were considerably greater in malignant tissues than in para-cancerous ones, consistent with the mRNA analysis. These results confirmed that ESCO2 was upregulated in various cancers, suggesting a promising future for ESCO2 in cancer diagnosis. Additionally, OS, DSS, DFI, and PFI analyses all revealed that ESCO2 was significantly associated with cancer patient’s prognosis. Evaluated ESCO2 resulted in poorer prognosis in ACC, BLCA, BRCA, CESC, COAD, GBM, HNSC, KIRC, LGG, LIHC, LUAD, LUSC, and PAAD. However, in THYM, COAD and READ, ESCO2 was a favorable prognostic factor. ESCO2 has been shown to prevent cancer metastasis in a recent trial on colon cancer, which is consistent with the results of our bioinformatics research [19]. According to the findings above, ESCO2 is crucial in determining a cancer patient’s prognosis and can serve as a reliable prognostic biomarker. To some extent, tumor grading and clinical staging can show how tumors progress. Our results show that ESCO2 expression was positively associated with tumor stage and size in LIHC, KIRC, KIRP and LUAD. Furthermore, ESCO2 was crucial for the metastasis of COAD, KIRC, and LUAD. Our research identified that ESCO2 is a proto-oncogene that is linked to the development and progression of tumors.

Emerging research has confirmed that ESCO2 participated in apoptosis and cell proliferation-related pathways, such as the P53 and mTOR pathway [15, 17]. Furthermore, ESCO2 could stimulate aerobic glycolysis in LUAD cells by upregulating PKM2 and downregulating PKM1 expression [16]. The GSEA results in our study were unexpectedly uniform among the 12 different cancers, indicating the validity of our findings. The cell cycle and proliferation-related E2F targets, G2M checkpoint, mitotic spindle, and Mtorc1 signaling were concentrated in the genes upregulated in the ESCO2-high group. These ESCO2-high patients also displayed greatly enhanced DNA repair. In sum up, our bioinformatics analysis confirmed ESCO2’s contribution to the control of cell proliferation and cycle. Therefore, we conducted experimental validation to verify the results of the bioinformatics analysis. We knocked down ESCO2 in A498 and T24 cells, and ESCO2’S knockdown significantly inhibited the proliferation, invasion, and migration of A498 and T24 cells. This result demonstrated that ESCO2’s role in cancer might be unified, further indicating the possibility that it could be an effective therapeutic target. Furthermore, we investigated its potential regulatory mechanisms. We found the core of the ESCO2-CDK1 regulatory network. When ESCO2 was inhibited, CDK1 expression decreased, demonstrating that CDK1 is a downstream gene controlled by ESCO2. Cell cycle progression is the central event in all proliferating cells and is primarily regulated by cell cycle-dependent kinases (CDK) [42].

CDK1 is the only CDK required for the G2-M transition and regulates G1 progression and G1-S transition [43]. In recent years, CDK1 has been suggested as a therapeutic target for cancer. CDK1 overexpression has been found in many cancers, including gastric cancer, ovarian cancer, oral squamous cell carcinoma, liver cancer, and breast cancer [44]. CDK1 inhibitors could be a potential small-molecule drug. CDK1 inhibitor RO3306 could improve the efficacy of sorafenib treatment by targeting cancer stem cells in a preclinical model of hepatocellular carcinoma [45]. In addition, Several CDK1 inhibitors, including Rigosertib (phase II/III) and Zotaraciclib, have begun phase I clinical trials for treating pancreatic cancer and glioma [46, 47]. As a CDK1’s upstream regulatory gene, ESCO2 took part in the control of the cell cycle. Hence, ESCO2 is likely to be a promising therapeutic target in the future. Finally, single-cell analysis observed that ESCO2 was expressed not only in malignant cells but also in endothelial and immune cells, especially proliferating T cells. The GSEA results also indicate that ESCO2 is involved in cancer inflammation control, including IL2 STAT5 signaling, which raises the possibility that ESCO2 also controls the tumor immune microenvironment. Studies have shown that inhibition of cell cycle protein not only induce tumor cell cycle arrest, but also promote anti-tumor immunity. Inhibition of CDK4 /6, for example, increases levels of PD-L1 protein. More excitingly, combining CDK4/6 inhibitor with anti-PD-1 immunotherapy promotes tumor regression and significantly increases overall survival in mouse tumor models [48]. Therefore, research into ESCO2’s function in the tumor immune microenvironment is essential.

This study has the following limitations, though. First, this work does not apply any unique clinical cohorts to evaluate the diagnostic and prognostic significance of ESCO2 in cancer, despite its extensive use of sequencing data from public databases. Second, this study only performed ESCO2’s functional experiment in ccRCC cells; the upstream and downstream pathways of ESCO2 were not comprehensively examined, and the precise molecular mechanism of ESCO2 regulation is still unknown. Third, we did not conduct experiments in vivo, which is an important issue. We should attempt to resolve this issue in the future.

In conclusion, ESCO2 is a potential biomarker and oncogene for pan-cancer that can accurately predict the cancer patient’s prognosis in ACC, KICH, KIRC, KIRP, LGG, LIHC, LUAD, MESO, PAAD, THYM, COAD, and READ. Furthermore, our bioinformatics results discovered that ESCO2 is involved in cell division and cell cycle regulation and verified that ESCO2 is essential for the proliferation and invasion of human ccRCC and BLCA cells in vitro. In a nutshell, ESCO2 is a potential and reliable therapeutic target.

### Electronic supplementary material

Below is the link to the electronic supplementary material.

Supplementary Material 1

Supplementary Material 2

Supplementary Material 3

Supplementary Material 4

## Data Availability

The data analyzed in this study can be downloaded from the GEO (GSE156632, https://www.ncbi.nlm.nih.gov/geo/) and TCGA (https://portal.gdc.cancer.gov/).

## Abbreviations

DEG: differentially expressed genes
ORR: objective response rate
QC: quality control
tSNE: t-Distributed Stochastic Neighbor Embedding
ROC: Receiver operating characteristic curve
GSEA: gene set enrichment analysis
KEGG: Kyoto Encyclopedia of Genes and Genomes
TME: tumor microenvironment
