# Supplementary material for: ESCO2’s oncogenic role in human tumors: a pan-cancer analysis and experimental validation
Source: BMC Cancer. 2024 Apr 11;24:452. doi: 10.1186/s12885-024-12213-w (PMC11007995; doi:10.1186/s12885-024-12213-w)
Supplement: Supplementary file 1 — Supplementary Material 1 [file 12885_2024_12213_MOESM1_ESM.docx]

**Figure S2 High expression of ESCO2 affects DSS in various malignancies**


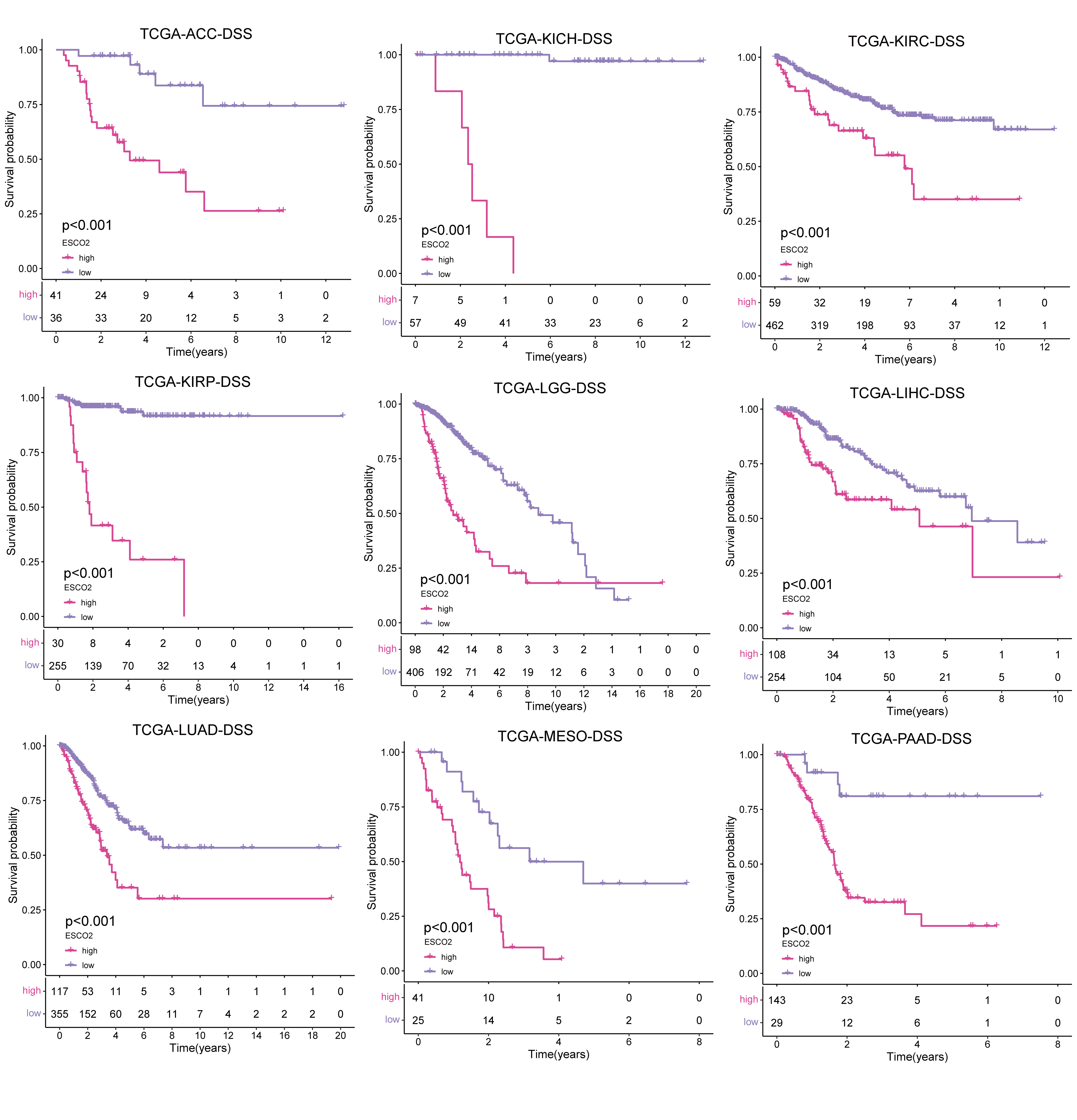


**Figure S2**

Kaplan–Meier analysis of the association between ESCO2 expression and DSS
