# Supplementary material for: ESCO2’s oncogenic role in human tumors: a pan-cancer analysis and experimental validation
Source: BMC Cancer. 2024 Apr 11;24:452. doi: 10.1186/s12885-024-12213-w (PMC11007995; doi:10.1186/s12885-024-12213-w)
Supplement: Supplementary file 2 — Supplementary Material 2 [file 12885_2024_12213_MOESM2_ESM.docx]

**Figure S3** **High expression of ESCO2 affects PFI in various malignancies**


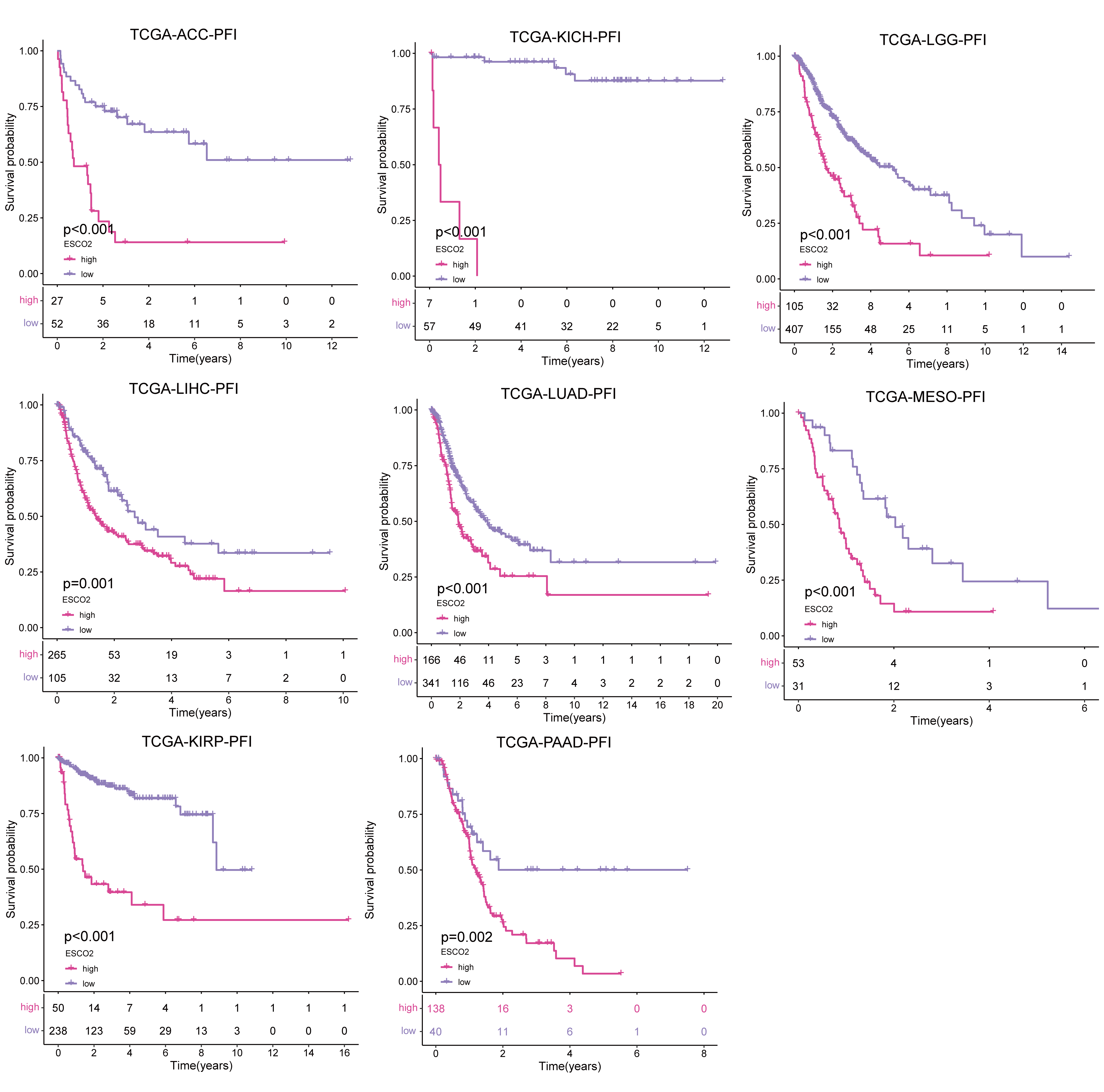


**Figure S3**

Kaplan–Meier analysis of the association between ESCO2 expression and PFI
