# Supplementary material for: ESCO2’s oncogenic role in human tumors: a pan-cancer analysis and experimental validation
Source: BMC Cancer. 2024 Apr 11;24:452. doi: 10.1186/s12885-024-12213-w (PMC11007995; doi:10.1186/s12885-024-12213-w)
Supplement: Supplementary file 3 — Supplementary Material 3 [file 12885_2024_12213_MOESM3_ESM.docx]

**Figure S1 ESCO2 has strong predicting ability for multiple malignancies.**


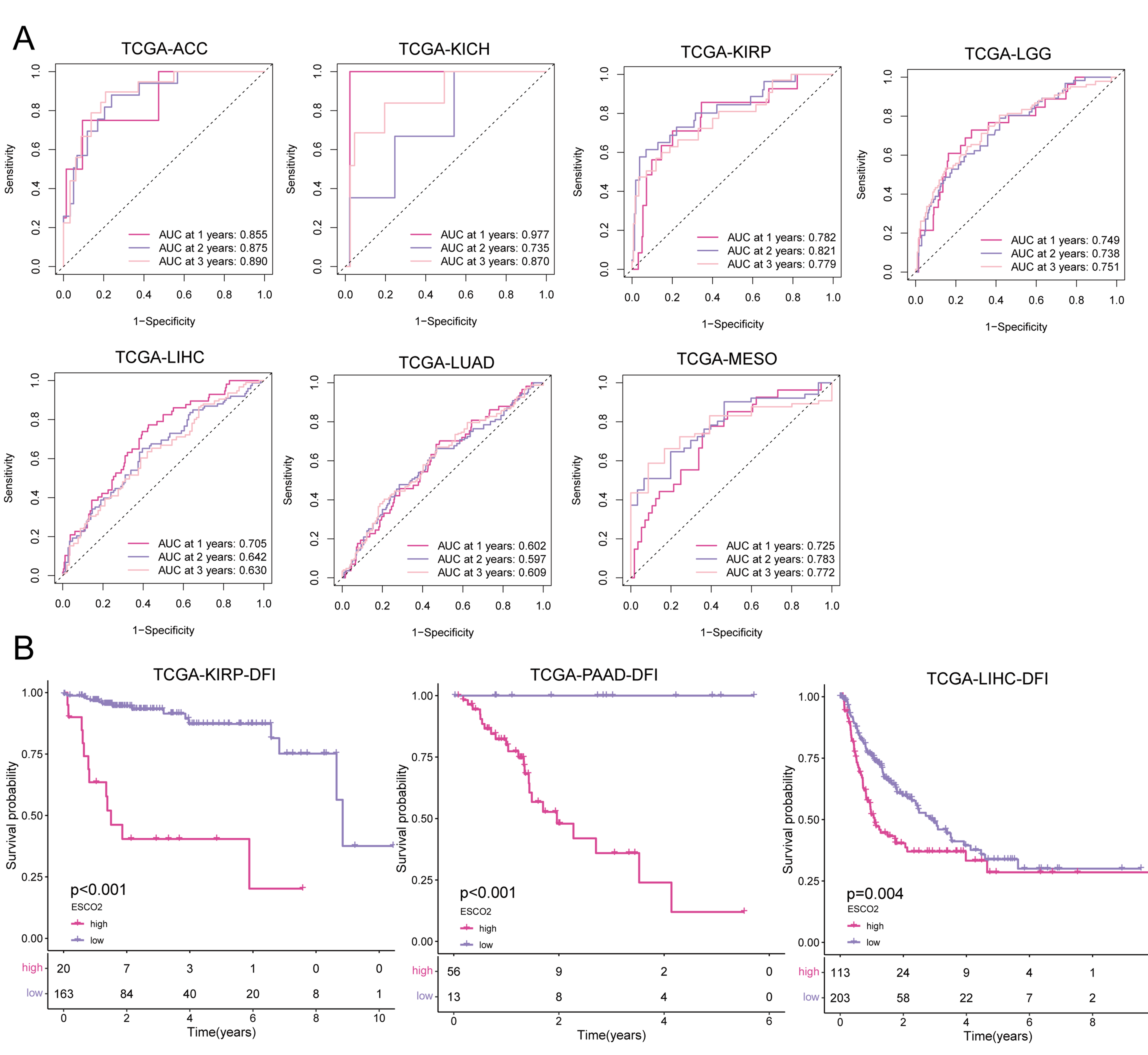


**Figure S1**

(A) Receiver operating characteristic curves of the ESCO2 expression to predict the 1-, 3-, and 5-year OS in ACC, KICH, KIRP, LGG, LIHC, LUAD and MESO.

(B) Kaplan–Meier analysis of the association between ESCO2 expression and DFI
