# Supplementary material for: ESCO2’s oncogenic role in human tumors: a pan-cancer analysis and experimental validation
Source: BMC Cancer. 2024 Apr 11;24:452. doi: 10.1186/s12885-024-12213-w (PMC11007995; doi:10.1186/s12885-024-12213-w)
Supplement: Supplementary file 4 — Supplementary Material 4 [file 12885_2024_12213_MOESM4_ESM.pptx]

## Slide 1
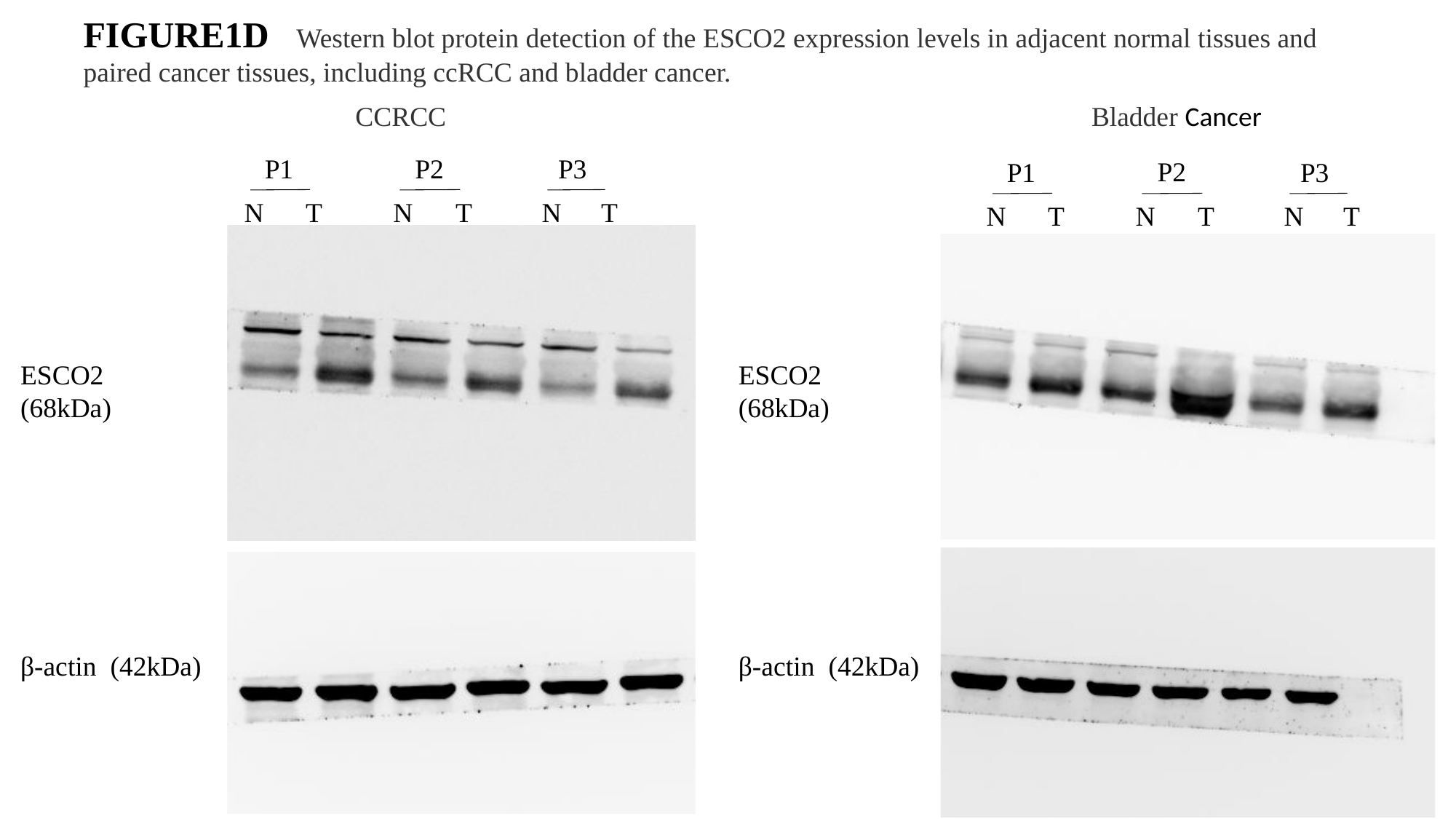

FIGURE1D Western blot protein detection of the ESCO2 expression levels in adjacent normal tissues and paired cancer tissues, including ccRCC and bladder cancer.
CCRCC
Bladder Cancer
P2
N
T
P1
N
T
P3
N
T
P2
N
T
P1
N
T
P3
N
T
ESCO2 (68kDa)
ESCO2 (68kDa)
β-actin (42kDa)
β-actin (42kDa)

## Slide 2
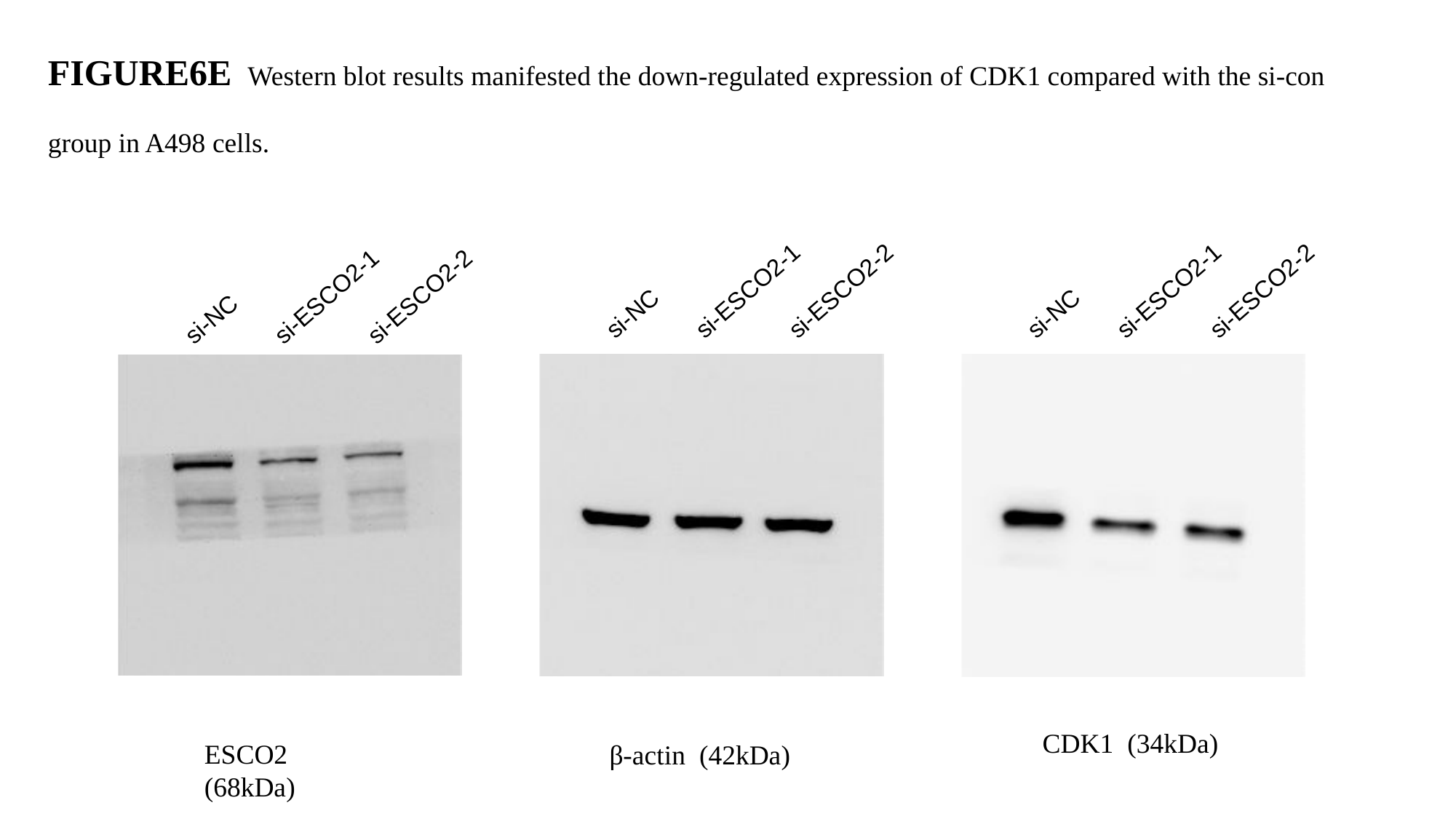

FIGURE6E Western blot results manifested the down‐regulated expression of CDK1 compared with the si‐con group in A498 cells.
si-ESCO2-1
si-ESCO2-2
si-ESCO2-1
si-ESCO2-2
si-ESCO2-1
si-ESCO2-2
si-NC
si-NC
si-NC
CDK1 (34kDa)
ESCO2 (68kDa)
β-actin (42kDa)

## Slide 3
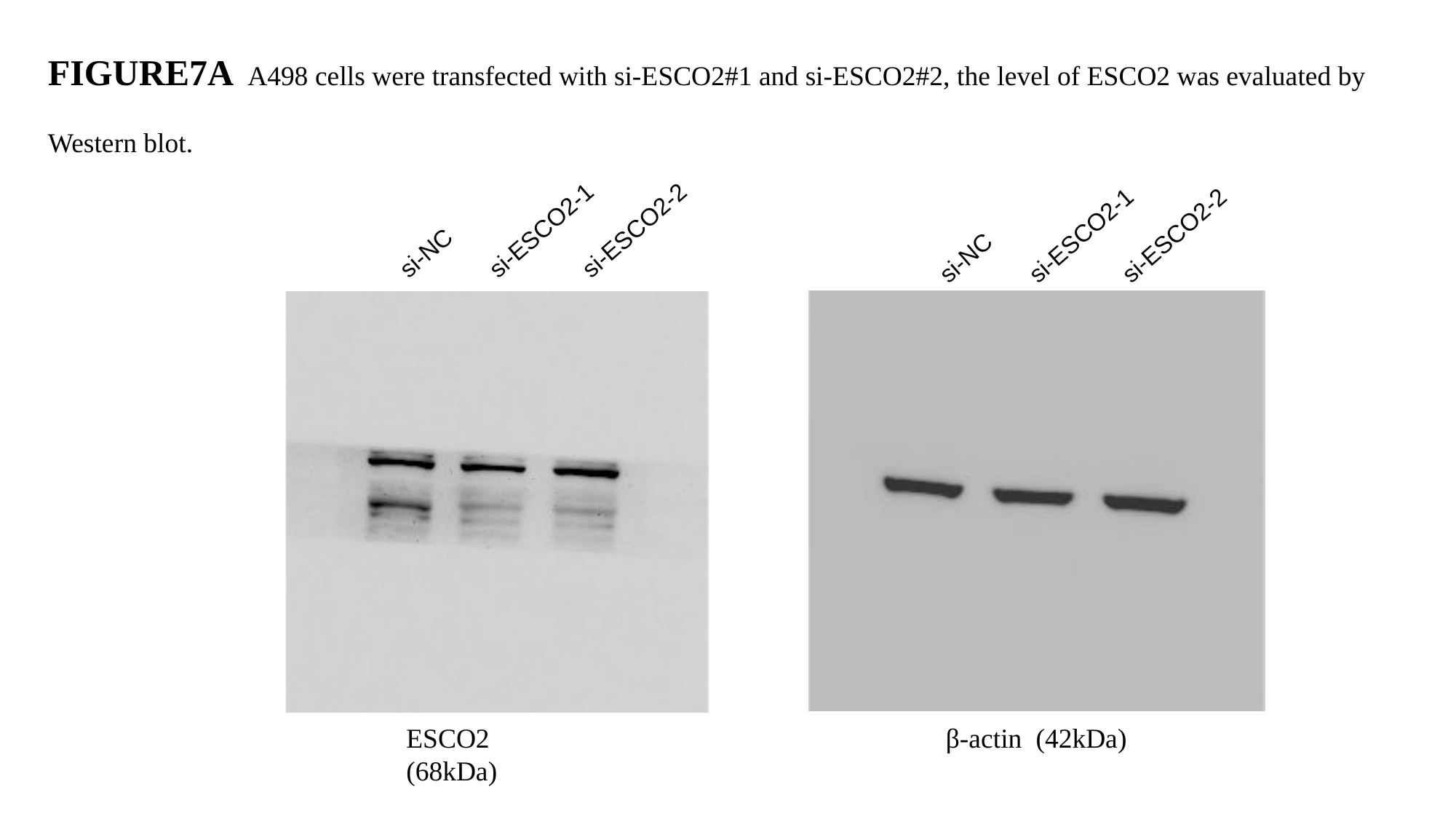

FIGURE7A A498 cells were transfected with si-ESCO2#1 and si-ESCO2#2, the level of ESCO2 was evaluated by Western blot.
si-ESCO2-1
si-ESCO2-2
si-ESCO2-1
si-ESCO2-2
si-NC
si-NC
β-actin (42kDa)
ESCO2 (68kDa)

## Slide 4
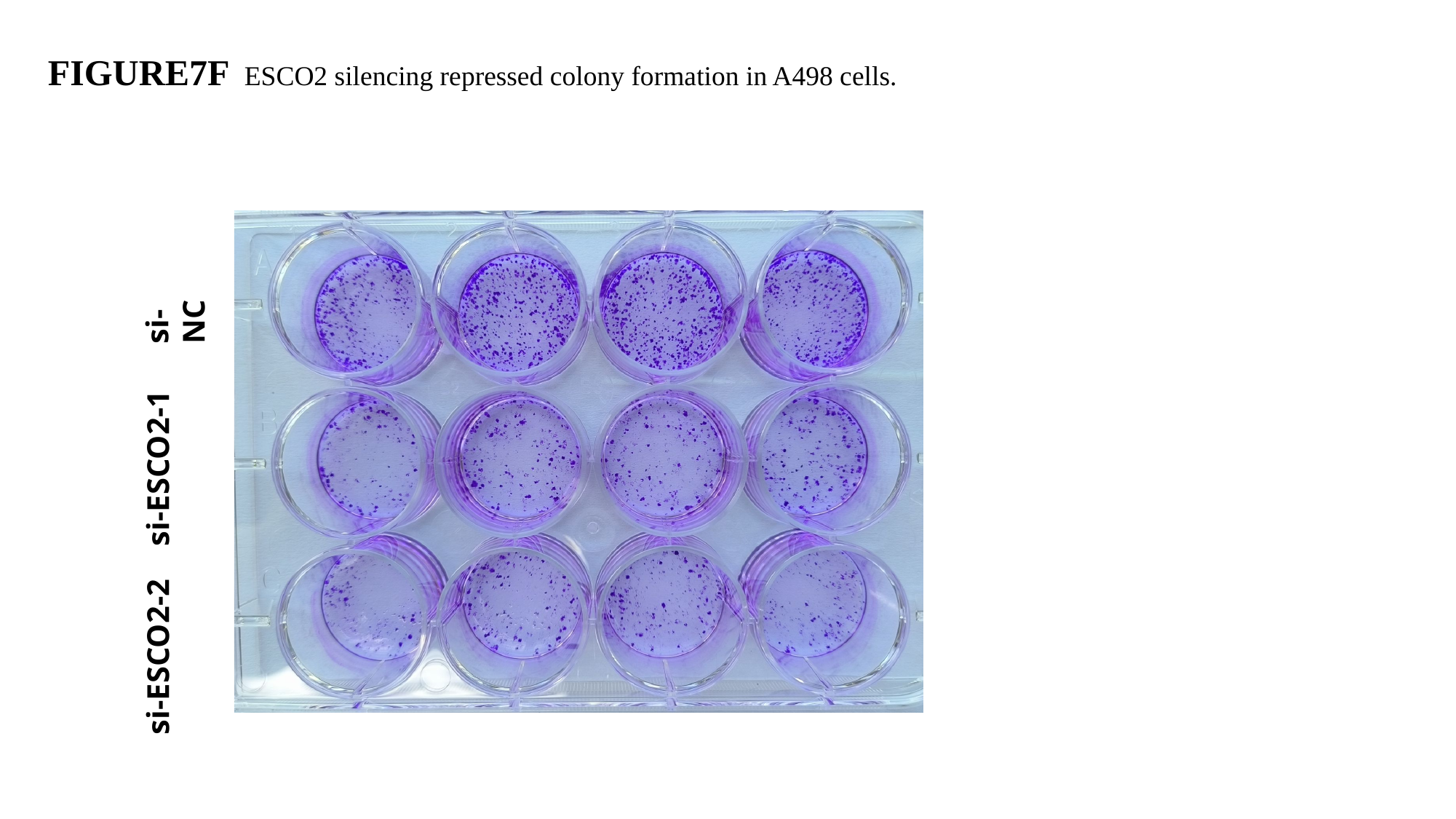

FIGURE7F ESCO2 silencing repressed colony formation in A498 cells.
si-NC
si-ESCO2-1
si-ESCO2-2

## Slide 5
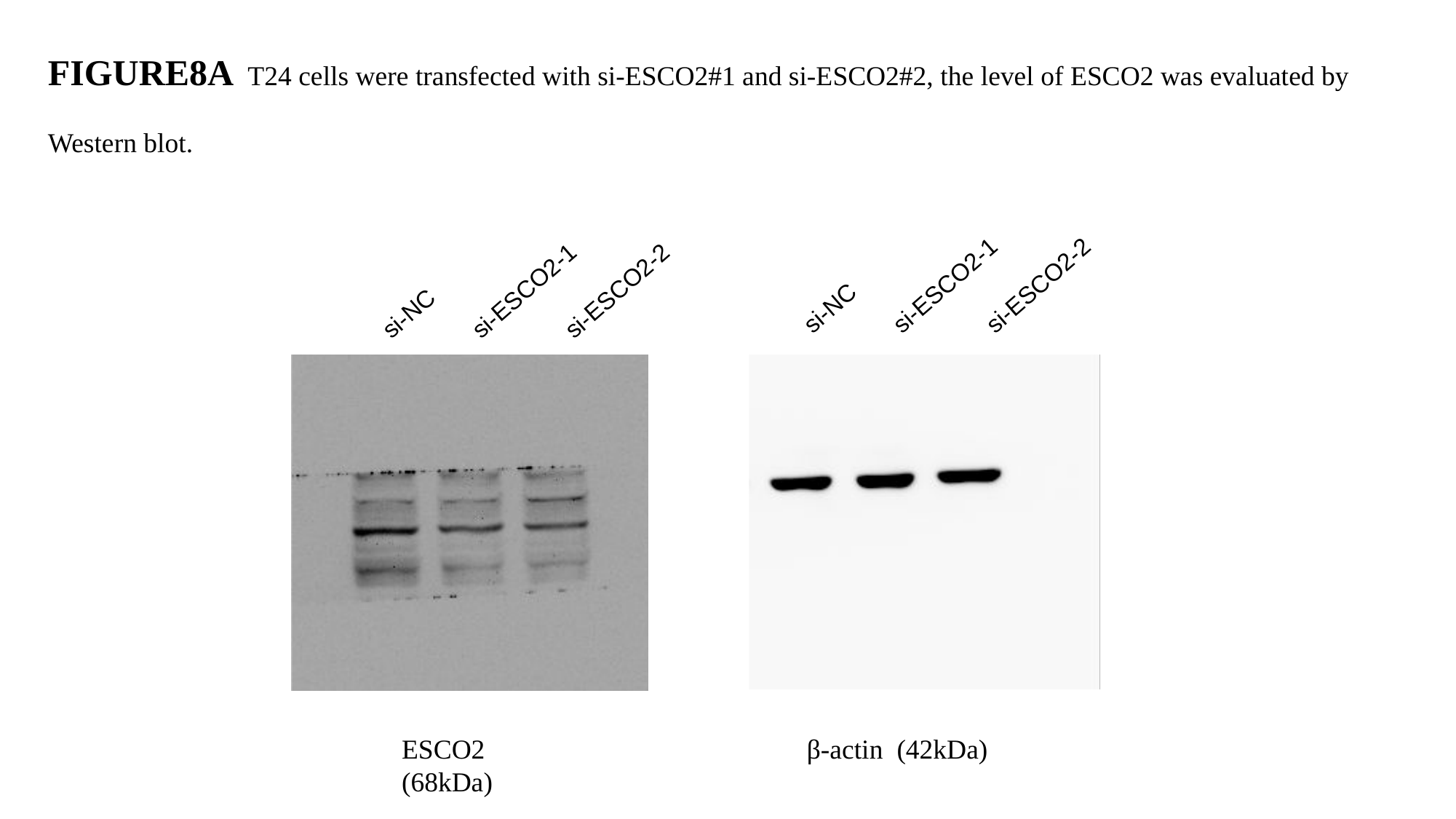

FIGURE8A T24 cells were transfected with si-ESCO2#1 and si-ESCO2#2, the level of ESCO2 was evaluated by Western blot.
si-ESCO2-1
si-ESCO2-2
si-ESCO2-1
si-ESCO2-2
si-NC
si-NC
ESCO2 (68kDa)
β-actin (42kDa)

## Slide 6
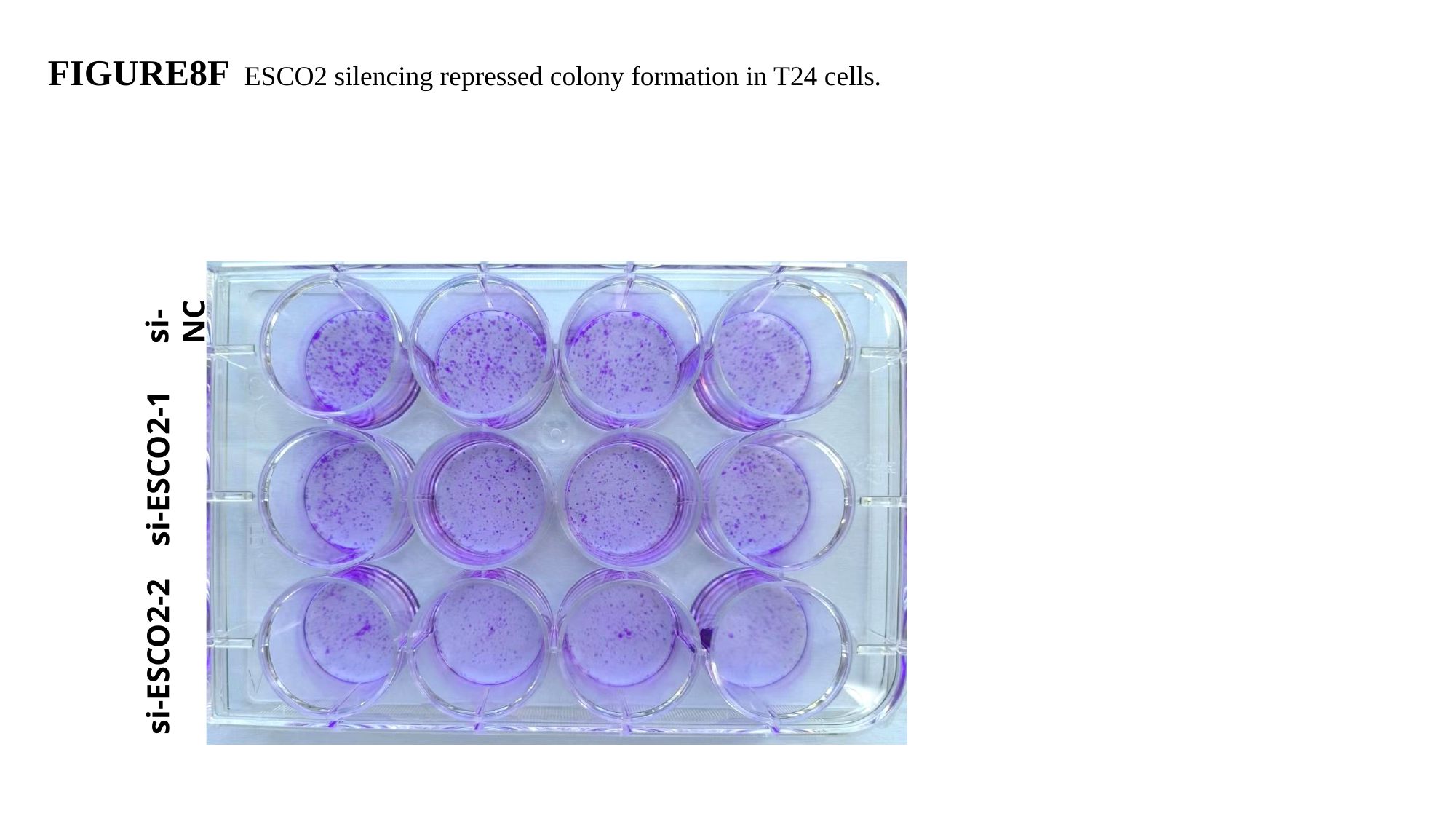

FIGURE8F ESCO2 silencing repressed colony formation in T24 cells.
si-NC
si-ESCO2-1
si-ESCO2-2
